# Supplementary material for: Hypoxia-driven remodeling of SELENOP+ macrophages shapes T cell dynamics and promotes ovarian cancer metastasis
Source: Nat Commun. 2026 Jan 12;17:1097. doi: 10.1038/s41467-025-67859-2 (PMC12852879; doi:10.1038/s41467-025-67859-2)
Supplement: Supplementary file 1 — Supplementary Information [file 41467_2025_67859_MOESM1_ESM.pdf]

Hypoxia-Driven Remodeling of SELENOP^{+} Macrophages Shapes T Cell Dynamics and Promotes Ovarian Cancer Metastasis

Supplementary Notes: 1
Supplementary Methods: 5
Supplementary Figures and Supplementary Figure Legends: 12
Supplementary References 39

Supplementary Notes:

Supplementary Note 1: Identification of major clusters

We identified major clusters using canonical marker expression: lymphocytes, including T cells (*CD3D*, *CD3E CD3G*, and *TRAC*), natural killer (NK) cells (*NCAM1*, *NCR1*, *KLRF1*, and *KLRC1*) as well as B cells and plasma cells *(CD79A*, *CD79B*, *IGHG1*, and *JCHAIN*); myeloid cells (*LYZ*, *AIF1*, *CD14*, and *FCGR3A*); epithelial cells (*WFDC2*, *PAX8*, *KRT18*, and *EPCAM*); stromal cells, including fibroblasts (*COL1A1*, *COL3A1*, *DCN* and *OGN*), cancer-associated fibroblasts (CAF) (*FAP*, *ACTA2*, *THY1* and *POSTN*), mesothelial cells (*UPK3B* and *LRRN4*), and smooth muscle cells (SMC)/Pericytes (*RGS5* and *MYH11*); and endothelial cells (*VWF*, *SPARCL1*, *FLT1* and *CLDN5*) (Fig. 1c,d and Supplementary Fig. 1a).

To assess tissue preference patterns and dynamic changes of major cell clusters during HGSOC metastasis, we performed a Ratio of Observed to Expected ($R_{o/e}$) analysis using our scRNA-seq data[1,2]. $R_{o/e}$ analysis has been widely used in single-cell transcriptomic studies to assess the enrichment of specific subpopulations across different sample types[1-4]. Notably, preferential enrichment of major cell clusters was observed between solid and fluid sites, EAT/LAT and Met (Fig.1e). Non-immune cells, including epithelial cells, fibroblasts, CAFs, SMCs/pericytes, and endothelial cells, were enriched within solid sites. In contrast, key immune cell populations, such as T cells, NK cells and myeloid cells, demonstrated a comparative enrichment in fluid sites (Fig. 1e, left). Among different solid sites associated with HGSOC metastasis, CAFs and mesothelial cells were preferentially observed in the Met group compared to the EAT and LAT groups (Fig.1e, right). Moreover, B/Plasma cells and myeloid cells were more abundant in the LAT and Met compared to the EAT tumor (Fig.1e, right). These findings highlight the differences in major cell clusters between the solid and fluid tumor microenvironments of HGSOC, and reveal dynamic changes in specific clusters within the solid tumor sites during metastatic progression, aligning with previous studies[4-6].

Supplementary Note 2: Identification of CD8$^+$ subclusters

Among the CD8$^+$ T cell clusters, we first identified three unconventional subpopulations, including CD8$^+$*GNLY*$^+$ NK-like cells (T05), CD8$^+$*SLC4A10*$^+$ mucosal-associated invariant T cells (MAIT) (T08), and CD8$^+$*TRDV2*$^+$ γδ T cells (T09) by canonical markers[7] (Fig. 2a and Supplementary Fig. 2b). These subpopulations were predominantly localized in fluid environments (Supplementary Fig. 2c, left). For conventional CD8$^+$ T cells, we identified distinct subsets based on transcriptional profiling. The T01_CD8T-CCR7 subcluster, characterized by high expression of *CCR7*, *SELL*, and *IL7R*, was classified as naive T cells (Tn). The T02_CD8T-GZMK subset, marked by elevated expression of *GZMK*, *DUSP2*, and *EOMES*, corresponded to effector memory T cells (Tem). T03_CD8T-XCL1, expressing *XCL1*, *XCL2*, and *ITGA1*, was identified as tissue-resident memory T cells (Trm) (Fig. 2a). Furthermore, the T07_CD8T-IFIT3 cluster, with high expression of *IFIT1*, *IFIT2*, and *IFIT3*, was identified as ISG (interferon-stimulated gene) T cells (Tisg) (Fig. 2a).

Supplementary Note 3: Identification of CD4$^+$ subclusters

The CD4$^+$ T-CCR7 subcluster, defined by high expression of *IL7R*, *CCR7*, and *TCF7*, was annotated as naïve T cells (Tn). The CD4$^+$ T-GZMK subcluster, marked by elevated expression of *GZMA*, *GZMH*, and *GZMK* and a high effector score, corresponded to effector memory T cells (Tem). The CD4$^+$ T-CXCL13 subset, characterized by high expression of *CXCL13*, *TOX*, and *PDCD1*, genes previously reported to be specifically enriched in Th1-like cells within the tumor microenvironment[8], exhibited an elevated Th1 score and was classified as Th1-like CD4$^+$ T cells. The CD4$^+$ T-FOXP3 subcluster expressed canonical regulatory T cell (Treg) markers, including *FOXP3*, *IL2RA*, and *CTLA4* was identified as Tregs. CD4$^+$ T-KLRB1 subset, with high expression of *KLRB1*, *CCR6*, and *RORA*, genes previously reported to be specifically enriched in Th17 cells within the tumor microenvironment[38], was classified as Th17-like CD4$^+$ T cells (Supplementary Fig. 2g-i and Supplementary Data 5). $R_{o/e}$ analysis revealed that both the CD4$^+$*CXCL13*$^+$ Th1-like and CD4$^+$*FOXP3*$^+$ Treg subpopulations were predominantly localized within the solid sites, whereas other

subsets were enriched in the fluid environments (Supplementary Fig. 2j, left).

Supplementary Note 4: Identification of myeloid subclusters

Based on canonical markers, subclusters M01–M04 were identified as dendritic cells (DCs) by high expression of *FCER1A*, *HLA-DQA1*, and *LILRA4*. M05 were monocyte-like cells with *VCAN*, *FCN1*, and *APOEC3A*. M06–M10 were classified as TAMs, marked by high expression of *CD68* and low expression of *FCN1* and *APOEC3A*. M11 was neutrophils (*FCGR3B* and *CSF3R*), M12 was mast cells (*CPA3* and *TPSB2* positive) and M13 was cycling cells (*STMN1* and *MKI67* positive), respectively (Fig. 3a, Supplementary Fig. 3a and Supplementary Data 5). Among the TAMs, five distinct subclusters were identified based on their gene signatures: *SELENOP*$^+$ macrophages (M06), *FN1*$^+$ macrophages (M07), *FABP5*$^+$ macrophages (M08), *SPP1*$^+$ macrophages (M09), and *CXCL10*$^+$ macrophages (M10) (Fig. 3a and Supplementary Data 5). The coexistence of M1 and M2 functional phenotypes within the TME of HGSOC underscores their high level of complexity, consistent with previous studies[9-11] (Supplementary Fig. 3b).

Supplementary Note 5: Hypoxia-related arm-level and RNA alterations

Our analysis revealed that subclones with high Hypoxia (MP6) signature scores exhibited specific genomic changes, including gains of 20q, 3q, 19q, 7q and 19p, as well as losses of 13q and 5q (Supplementary Fig. 12a). Building upon the established connections between the hypoxia-induced functional state of malignant cells and the metastatic progression as outlined earlier, we sought to further elucidate the molecular mechanisms underpinning hypoxia-driven metastasis. To this end, we conducted a detailed analysis of the dynamic alterations in the top-scoring 50 genes within the MP6 module across various groups. Among these 50 genes, nine were upregulated in the LAT and Met groups, with the highest expression observed in the Met group in our scRNA-seq data (Supplementary Fig. 12b and Supplementary Data 8). Furthermore, three (*FTL*, *EIF1,* and *PLIN*) out of the nine genes presented higher expression in metastatic lesions compared to adnexal tumors in our independent bulk RNA-seq

dataset[6] (Supplementary Fig. 12c), with poorer overall survival (OS) in OC patients (Supplementary Fig. 12d), and positive correlation with MP6 signature scores (Supplementary Fig. 12e). After culturing ovarian cancer cell lines under hypoxic conditions, ferritin light chain (*FTL*) mRNA expression increased (Supplementary Fig. 12f,g). *FTL* warrants further investigation in the context of HGSOC progression.

Supplementary Note 6: TME differences of *BRCA*-mutated patients

Whole-exome sequencing (WES) was performed to profile gene mutations in three patients. We focused on the homologous recombination repair (HRR)-associated genes[12]. Specifically, no damaging mutations in *BRCA1* or *BRCA2* were detected in two patients. However, damaging mutations were identified in *MRE11* of one patient, suggesting a HRD phenotype (Supplementary Data 9). Seven of eight patients (Supplementary Data 1) in our scRNA-seq cohort were HRD-positive. Then, six late-stage patients were subsequently classified into *BRCA* wild-type (BRCAw) and *BRCA* mutant (BRCAmut) groups. Our analysis revealed a relative enrichment of $CD8^+$*CXCL13*$^+$ Tex (T06) and $CD8^+$*GZMH*$^+$ Tpex (T04) in the BRCAmut group (Supplementary Fig. 16a). Notably, these cells exhibited enhanced cytotoxic signature scores in the BRCAmut group compared to the BRCAw group (Supplementary Fig. 16b). For macrophages, *SELENOP*$^+$ macrophages were preferentially detected in the BRCAmut group (Supplementary Fig. 16c), characterized by higher proinflammatory function score (Supplementary. Fig. 16d).

Supplementary Methods:

Supplementary Methods 1: Tissue preparation for spatial transcriptomic experiment

A tissue block with an edge length of less than 1 cm was dissected from the surgically removed tissues. The tissue block was then rinsed with cold PBS, immersed in a pre-cooled tissue storage solution (Miltenyi Biotec, Germany), and embedded with pre-cooled OCT (Sakura, USA) in a -30°C microtome (Thermo Fisher, USA) within 30 min after surgery. Three to four serial cryosections of 10 μm thickness were cut from the OCT-embedded samples for H&E staining, Stereo-seq library preparation, and IHC staining. Brightfield images of the H&E samples were obtained using a Motic microscope scanner (Motic, China) for histopathological assessment.

Supplementary Methods 2: Quality control of RNA obtained from OCT-embedded samples

Briefly, 100–200 μm thick sections were cut from each OCT-embedded sample for total RNA extraction using the RNeasy Mini Kit (Qiagen, USA) according to the manufacturer's protocol. RNA integrity number (RIN) was determined using a 2100 Bioanalyzer (Agilent, USA). Only samples with RIN≥7 qualified for the transcriptomic study. All samples had an RIN of 7–10.

Supplementary Methods 3: Stereo-seq library preparation and sequencing

The spatial transcriptomic RNA library was constructed using Stereo-seq capture chips (BGI-Shenzhen, China) with a size of 1 cm^2. The capture spots were 220 nm in diameter, with a center-to-center distance of 500 nm. Each Stereo-seq capture probe contained a 25 bp coordinate identity barcode, a 10 bp molecular identity barcode, and a 22 bp polyT tail for *in situ* mRNA hybridization. A cryosection of 10 μm thickness cut from OCT-embedded tissue was quickly placed on the chip, incubated at 37 °C for 3 min, and then fixed in pre-cooled methanol at -20 °C for 40 min. The fixed tissue sections were stained with Qubit ssDNA dye (Thermo Fisher, USA) to check the tissue integrity

before fluorescent imaging. The tissue sections were then permeabilized using 0.1% pepsin (Sigma, USA) in 0.01 N HCl buffer, incubated at 37 °C for 14 min, and then washed with 0.1× SSC. RNA released from the permeabilized tissue was reverse-transcribed for 1 h at 42°C. The tissue sections were then digested with a tissue removal buffer at 42 °C for 30 min. The cDNA-containing chip was then subjected to cDNA-release enzyme treatment overnight at 55 °C. The released cDNA was further amplified using a cDNA HIFI PCR mix (MGI). Approximately 20 ng of cDNA was fragmented to 400–600 bp, amplified for 13 cycles, and purified to generate a DNA nanoball library, which was sequenced with the single-end 50+100 bp strategy on an MGI DNBSEQ sequencer (MGI, China).

Supplementary Methods 4: Preliminary processing of Stereo-seq data

Stereo-seq raw data were automatically processed using the BGI Stereomics analytical pipeline (http://stereomap.cngb.org/), in which the reads were decoded, trimmed, deduplicated, and mapped against human GRCh38 genomes. Spots on the chip area covered by tissue were extracted based on the ssDNA and H&E staining images using the Lasso function of the BGI Stereomics website. To fully reflect the spatial transcriptomic landscape around the tumor areas, a bin size of 50 (50 spots × 50 spots, i.e., 24.86 × 24.86 μm) was used as the analytical unit. We filtered spots with UMIs < 20 and > 35000 as well as percentage of mitochondria genes > 0.25. We filtered out genes that were detected in less than 0.1% of all spots. Finally, 18807 protein-coding genes and 2102308 spots were used in ST analysis. For the validation cohort, data from GSE203612 processed through the SpaceRanger pipeline was downloaded and we removed spots with mitochondrial gene percentages greater than 20%; data from Licaj *et al* [13] that had undergone quality control by the authors was downloaded. For both datasets, we further utilized the filter_genes function of cell2location (version 0.1.0) to filter genes for subsequent deconvolution analysis. The filtering criteria were as follows: 1. The gene must have a count greater than 0 in at least 5% of the cells. 2.The gene must have a mean expression greater than 1.1 and a count greater than 0 in at least 0.05% of the cells.

Supplementary Methods 5: Spatial transcriptome

In the spatial transcriptome analysis of the discovery and validation cohort, we utilized cell2location with default settings to assess the spatial distribution of cell types. For each spatial transcriptome cohort, solid tumor scRNA-seq datasets from our single-cell atlas served as a reference. Next, for stereo-seq data, we deconvolute ST spots with hyper-parameters N_cells_per_location = 4 (bin50: 24.86 × 24.86 μm, approximately 3~4 cells), detection_alpha = 20, batch_size = 2048 and epoch = 1500. We used the "q05_cell_abundance_w_sf" output as the estimation of cell abundance in each spot as suggested. Spots were defined for a certain cell type by the largest value in "q05_cell_abundance_w_sf". For the deconvolution of the discover ST cohort, it was performed using cell2location with default parameters to quantify the spatial distribution of cell types. The q05_cell_abundance_w_sf output was used as the estimation of cell abundance in each spot, as suggested. We then applied spot-wise Pearson correlation with estimated cell type abundance to evaluate spatial colocalization patterns, similar to previous studies. A high positive Pearson correlation indicated similar spatial distributions between two cell types, while a negative correlation suggested distinct spatial distributions.

Supplementary Methods 6: Spatial neighborhood detection

We used MISTy's implementation in mistyR (version 1.8.1) to estimate the importance of the abundance of each cell type in explaining the abundance of the other major cell types[14]. The cell2location estimation for each slide were trained in a multi-view model using three different spatial contexts as the previous report[15]: (1) an intrinsic view that measures the relationships within a spot, (2) a juxta view that sums the observed deconvolution estimations of immediate neighborhood (largest distance threshold = 4, approximate 100 μm), and (3) a para view that weights the deconvolution estimations of more distant neighborhoods of each cell type (effective radius = 20 spots, approximate 500 μm). The aggregated estimated importance of each view of all slides were interpreted as cell-type dependencies in different spatial contexts, such as

colocalization or mutual exclusion. It should be noted that the reported interactions did not imply any causal relation. To associate microenvironment with certain epithelial subtypes, we fitted a MISTy model to explain the distribution of tumor subtypes with other cell types. The predictors were cell types except for epithelial cells, and the targets were weighted epithelial program scores (Eq. 1).

$$EPIsubtype \sim intra(EPIsubtype) + intra(TME) + juxta(TME) + para(TME) \tag{1}$$

For spatial cell-cell interaction analysis, we used the plot_interaction_communities function from mistyR to visualize predicted neighborhoods in a para view.

Supplementary Methods 7: Whole exome sequencing and variants filtration

Total genomic DNA was extracted from tumor tissues, quantified using Qubit dsDNA HS assay (Cat#N608301, Sangon), and assessed for integrity by 1% agarose gel electrophoresis. Next, the library preparation was performed using Covaris (Woburn, USA), Hieff NGS®MaxUp II DNA Library Prep Kit for Illumina® (Cat#13577ES96, YEASEN), SSELXT Human All Exon V6 (Cat#5191-6874, Agilent). Then the libraries were pooled and loaded on DNBseq-T7 (BGI, China) sequencer by 2×150 bp paired end sequence kit according to the instructions. Raw reads containing adaptor sequences and those with ambiguous or low-quality bases at the beginning or end were trimmed using Fastp. The qualified reads from each sample were aligned to the assembled human reference genome (hg38) using BWA (version 0.7.17) with default parameters. Single nucleotide polymorphisms (SNPs) and insertions-deletions (InDels) were called using Haplotype Caller of Genome Analysis ToolKit (GATK, version 4.1.2) for germline or MUTECT2 for somatic detection. ANNOVAR (20191024) was used to annotated the SNPs and InDels. The variants involved in homologous recombination repair (HRR)-associated genes were filtered with the following criteria: (1) variants classified as frameshift and nonsynonymous in tumor samples; (3) variants with minor allele frequency less than 0.04% in the human population (gnomAD_genome_EAS,

ExAC_EAS and esp6500); (4) variants predicted to be deleterious in at least three of five in silico analysis (PROVEAN, Polyphen2_HDIV, Polyphen2_HVAR, SIFT, and CADD), of which the criterion for CADD was greater than 20.

Supplementary Methods 8: Flow cytometry

Cells grown in vitro from each group were first stimulated with the cell stimulation cocktail (Cat#00-4975-93, Invitrogen) for 5 h. Next, cells were stained with eBioscience™ Fixable Viability Dye eFluor™ 780 (Cat#65-0865-14, Invitrogen) or Zombie Red™ Fixable Viability Kit (Cat#423109, Biolegend), blocking with anti-mouse CD16/32 (Cat#101320, BioLegend), or Human TruStain FcX™ (Cat#422302, Biolegend), and staining for 30 min at 4℃. Cells were stained with the anti-mouse/human antibody and fixed/permeabilized using the Cytofix/Cytoperm™ Fixation/Permeabilization Kit (Cat#554714, BD Biosciences). Cells were resuspended in PBS with 1% FBS for flow cytometry analysis. Cells were analysed using a FACS Fortessa flow cytometer (BD FACSymphony A1) equipped with appropriate lasers and filters. FACS Diva Software (v8.0.2, BD) was conducted to collect FACS data. Data were analysed using FlowJo (versions V10.8.1). Appropriate gating strategies were applied to exclude debris and ensure accurate analysis of single cells. Antibodies used in flow cytometry are as follows: anti-CD45.1 monoclonal antibody (0.5 μg/test, Cat#25-0453-81, Thermo), anti-CD45.2 antibody (0.25 μg/test, Cat#147716, Biolegend), anti-CD3e monoclonal antibody (0.5 μg/test, Cat#48-0031-82, Invitrogen), anti-CD8a monoclonal antibody (0.5 μg/test, Cat#69-0081-82, Invitrogen), anti-F4/80 antibody (0.5 μg/test, Cat#416-4801-82, Thermo), anti-CD11b antibody (0.5 μg/test, Cat#550993, BD Pharmingen), anti- GZMB monoclonal antibody (1 μg/test, Cat#25-8898-82, Invitrogen), anti- GZMB monoclonal antibody (1 μg/test, Cat#372208, BioLegend), anti-PRF1 monoclonal antibody (1 μg/test, Cat#154304, Biolegend), anti-PRF1 monoclonal antibody (1 μg/test, Cat#17-9392-80, Invitrogen), anti-PD-1 antibody (1 μg/test, Cat#135225, Biolegend), anti-SPP1 antibody (1 μg/test, Cat#sc-73631PE, SantaCruz), anti-SELENOP antibody (1 μg/test, Cat#sc-376858FITC, SantaCruz), anti-CD86 monoclonal antibody (0.5 μg/test, Cat#63-0862-80,

Invitrogen), anti-CD206 monoclonal antibody (0.5 μg/test, Cat#17-2061-80, Invitrogen). Annexin V-FITC/PI cell apoptosis detection kit (Bestbio, BB-4101) was used to detect the apoptosis rate of ID8-OVA-luciferase cells and OVCAR3. The detail gating strategy for sorting the the target cells is shown in the Supplementary Fig. 17.

Supplementary Methods 9: Western blot

A mixture with 25 μg of protein and 5×loading buffer was added to 10% SDS-polyacrylamide gel for electrophoresis and transferred to polyvinylidene fluoride (PVDF) membrane (BS-PVDF-22, Bioshop). After sealing, the cells were incubated overnight with anti-GPX1 antibody (1:1000, Cat#T56586S, Abmart), anti-SELENOP antibody (1:900, Cat#PA5-112707, Invitrogen), anti-SELENOP antibody (1:1000, Cat#sc-376858, SantaCruz), anti-SELENOP antibody (1:1000, Cat#ab277526, Abcam), anti EPHB2 antibody (1:1000, Cat#83277-1-RR, Proteintech), anti-VEGFA antibody (1:1000, Cat# 512810, BioLegend) at 4°C for 16 h. The membrane was coupled with goat anti-rabbit IgG-HRP antibody (1:5000, Cat# abs20039, Absin) at room temperature for 1 h. The relative protein content was detected using ImageJ software density method. anti-GAPDH antibody (1:5000, Cat#60004-1-Ig, Proteintech), anti- β-actin antibody (1:5000, Cat#66009-1-Ig, Proteintech) were used as the loading control. The uncropped and unprocessed scans of the blots are provided as a Source Data file.

Supplementary Methods 10: RNA isolation and RT-qPCR

We used TRIzol Reagent (Cat#15596026CN, Thermo Fisher Scientific) to isolate Total cellular RNA. PrimeScript™ RT reagent Kit (Cat#RR037A, Takara) was used to reverse total RNA transcribed into cDNA. Quantitative reverse transcription PCR was performed by using TB Green Premix Ex Taq™ II (Cat#RR820A, Takara) and gene-specific primers with expected to yield an amplicon of 87 bp for FTL and 275 bp for β-actin. The qPCR primers sequences were as follows: FTL-forward: 5′-TCCGATTTCCTCTCCGCTTG-3′, FTL-reverse: 5′-AACCACAAAAACGGTGCTGG-3′, β-actin-forward: 5′-GGGAAATCGTGCGTGACATTAAG-3′, β-actin-reverse: 5′-

TGTGTTGGCGTACAGGTCTTTG-3′. The dissociation curves were also run routinely to ensure specificity of reactions. The amplified products were checked by agarose gel (1.5%) electrophoresis.

Supplementary Figures and Supplementary Figure Legends:

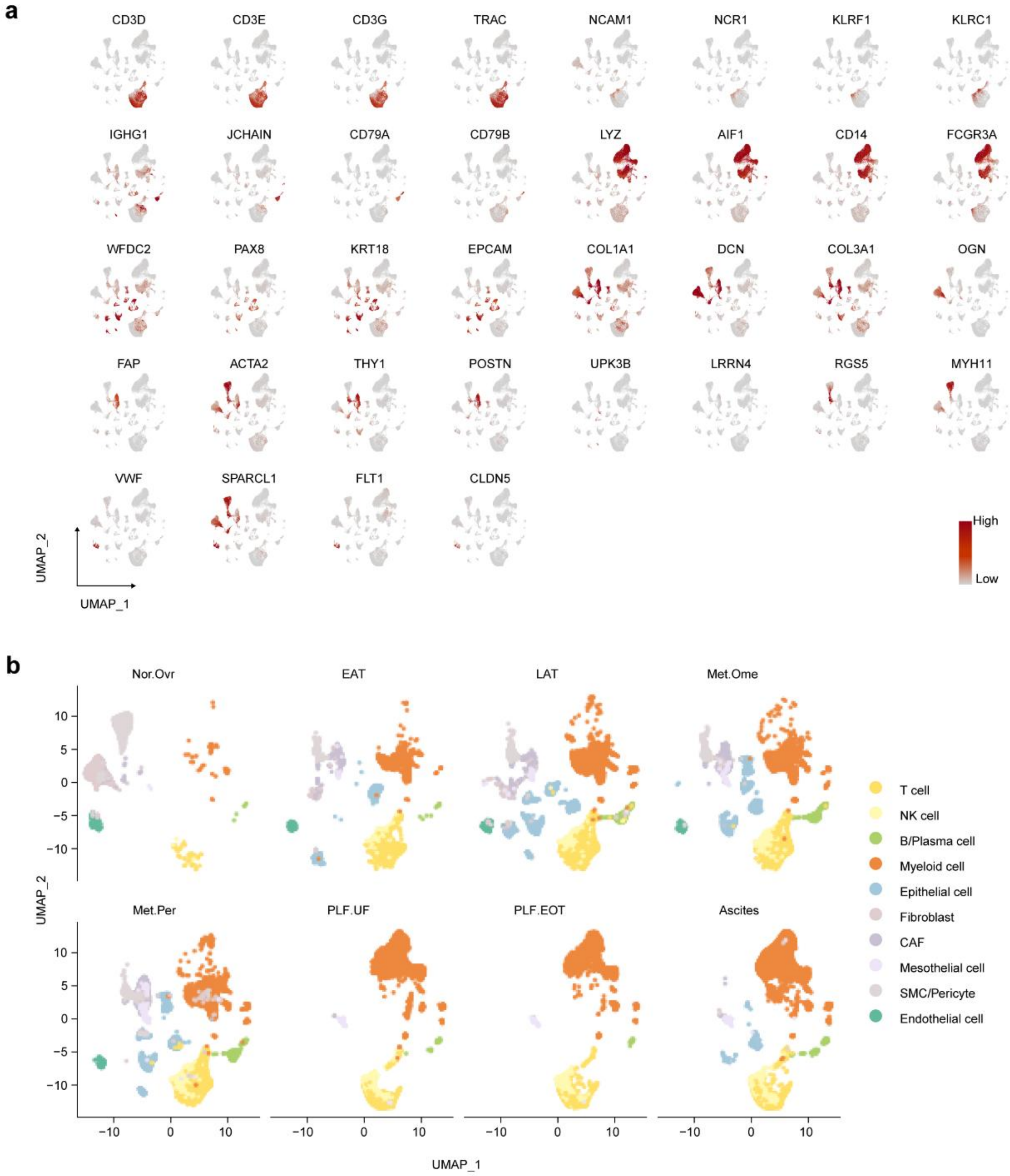

Supplementary Fig. 1 Dissection and dynamic changes of scRNA-seq atlas associated with HGSOC metastasis. a, UMAP plots showing the expression of marker genes for cell types annotation. **b**, UMAP plots illustrating the distinct cell composition of different groups, colored by corresponding main cell type colors in Fig.1c. For **a**, **b**, n = all 34 scRNA-seq cohort samples, biological replicates.

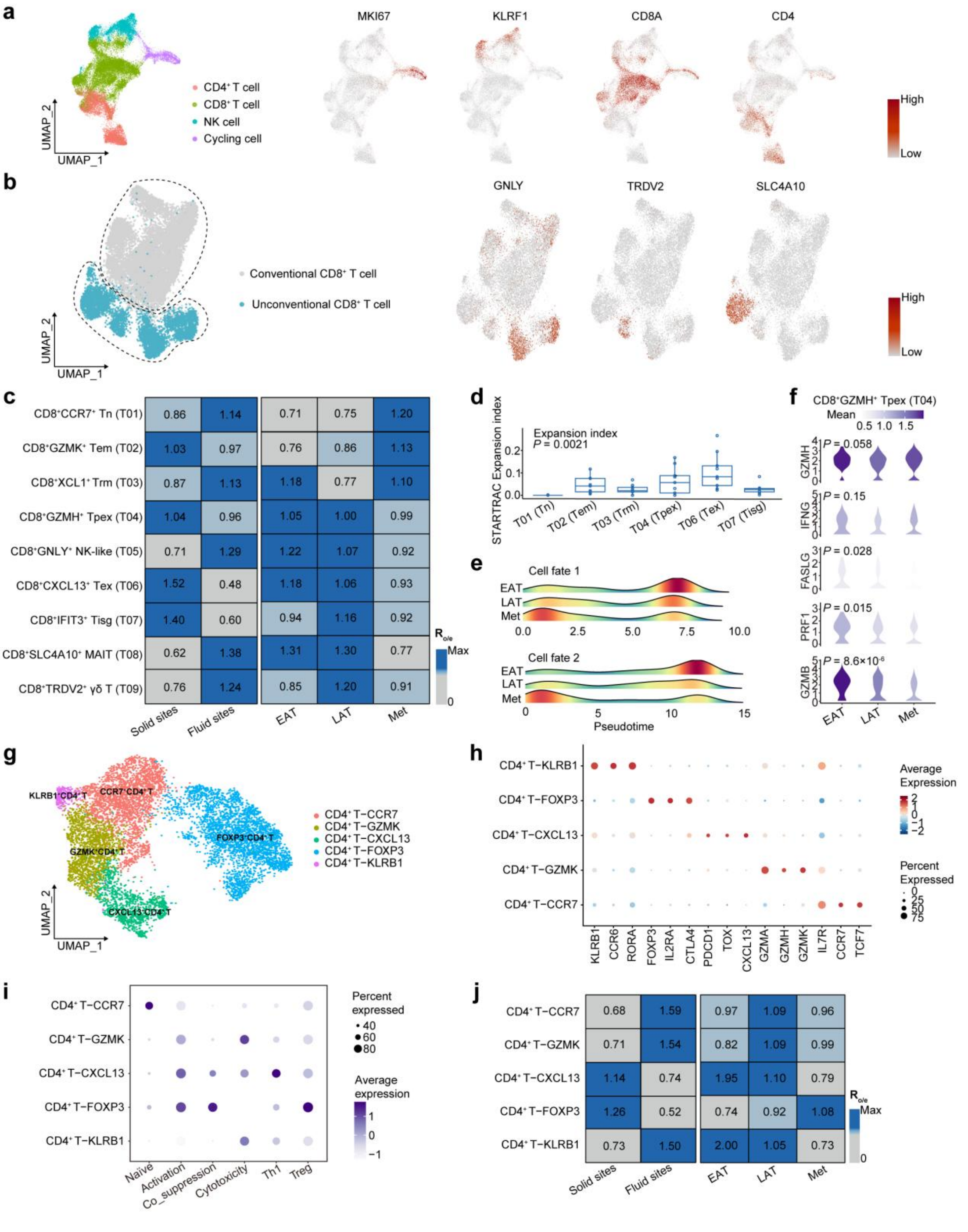

Supplementary Fig. 2 Dissection of the characteristics and dynamics of T cells in the tumor microenvironment associated with HGSOC metastasis. a, UMAP plots depicting the clusters of all T/NK cells, colored by cluster (left) and UMAP plots showing the expression of marker genes (right). **b**, UMAP plots representing conventional and unconventional $CD8^+$ T cells with distinct clusters highlighted by black dotted lines (left) and UMAP plots showing the expression of marker genes for unconventional $CD8^+$ T cells (right). **c**, Tissue preference of each $CD8^+$ T cluster, estimated by $R_{o/e}$ (the ratio of observed to expected cell numbers) ($P = 2.2\times10^{-16}$, left) ($P = 3.512\times10^{-15}$, right). *P*-values are calculated by the two-sided chi-squared test. **d,** Clonal expansion of the clusters of conventional $CD8^+$ T cells quantified by STARTRAC indices (HRA002184). Each dot represents a

patient. Center line indicates the median value, lower and upper hinges represent the 25th and 75th percentiles, respectively and whiskers denote 1.5 × interquartile range. **e,** Density plot showing the density patterns of cells from different tumor sites along the cell fate 1 (upper) and cell fate 2 (lower). **f,** Violin plot showing cytotoxic factors of T04 across different groups, colored by average expression. **g,** UMAP plots depicting the clusters of all $CD4^+$ T cells, colored by cluster. **h,** Dot plot showing marker genes of $CD4^+$ T clusters. **i,** Dot plot showing expression patterns of functional signatures across indicated clusters. **j**, Tissue preference of each $CD4^+$ T clusters, estimated by $R_{o/e}$ (P = 2.2×10^{-16}). P-values are calculated by the two-sided chi-squared test. For **a, b**, **g, h, i,** n = 34 scRNA-seq cohort samples, biological replicates. For **d**, a total of n = 14 HGSOC samples, biological replicates. For **e, f**, n = 17 scRNA-seq cohort solid site samples, biological replicates. For **c**, **j**, n = 25 scRNA-seq cohort samples except for Nor.Ovr and PLF.UF, biological replicates. For **d, f,** P-values are calculated by the two side Kruskal−Wallis test with Bonferroni post hoc test. Source data are provided as a Source Data file.

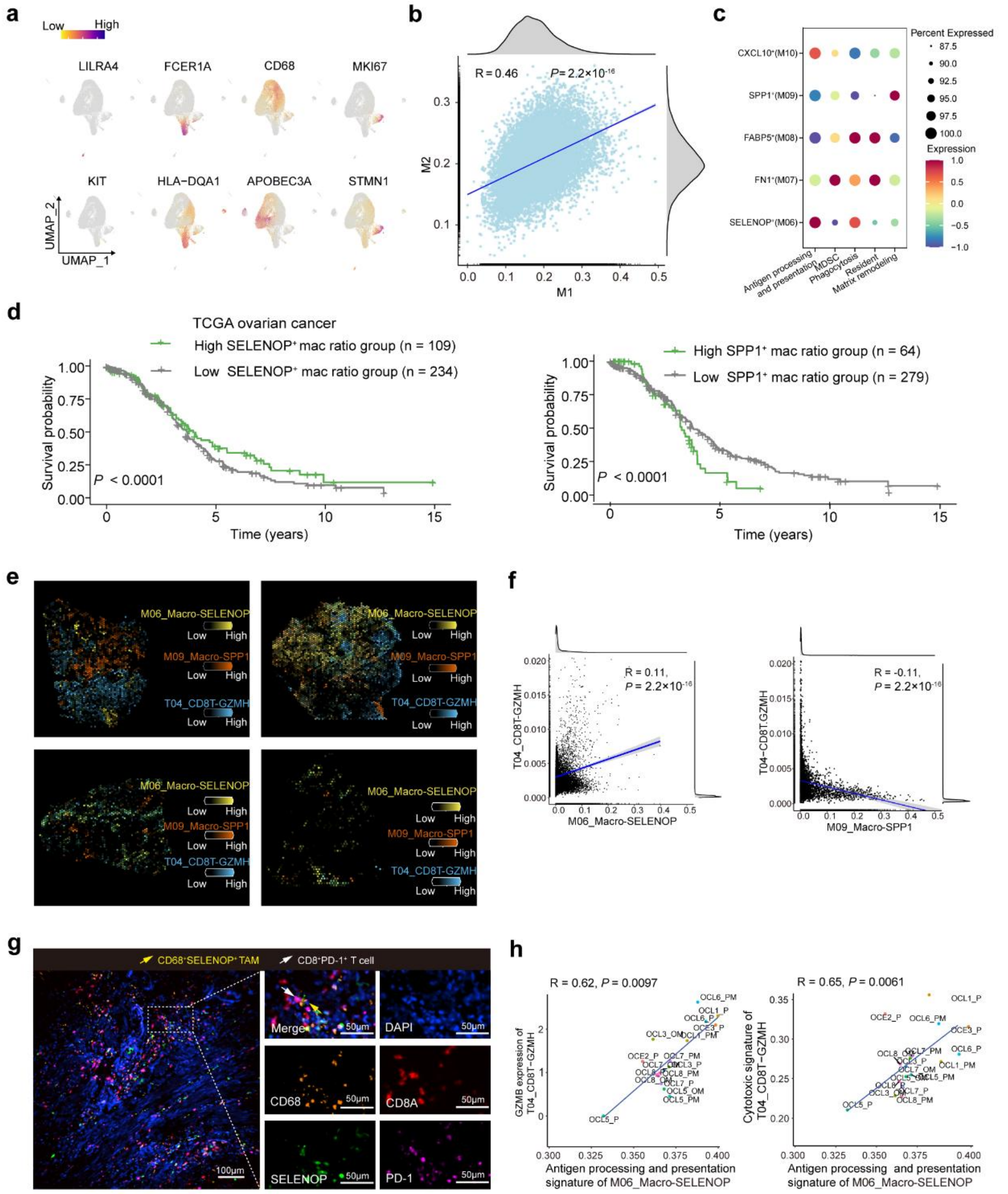

Supplementary Fig. 3 Dissection of the characteristics of macrophages in the tumor microenvironment associated with HGSOC metastasis. a, UMAP plots showing the expression of marker genes for myeloid clusters. **b**, Scatterplot showing the Spearman correlation of the M1 score and M2 score of macrophages. **c,** Dot plot showing expression patterns of functional signatures across indicated clusters. Dot size represents percent of expressing cells in each cluster and color represents z-score of normalized mean expression level of selected genes. **d**, The Kaplan–Meier overall survival curves of patients with HGSOC grouped by proportion of *SELENOP*$^{+}$ macrophage (left) and *SPP1*$^{+}$ macrophage (right). *P*-values were determined by log-rank test. **e**, Representative spatial co-localization of *SELENOP*$^{+}$ macrophages with T04_CD8T-GZMH in tumor spots of slide from adnexal sites in the spatial RNA validation cohort (n = 5). The cell abundances were estimated by cell2location. **f**, Scatterplot showing the Pearson correlation between

SELENOP$^+$ macrophages (left) or *SPP1*$^+$ macrophages (right) and T04_CD8T-GZMH abundance (by cell2location) in spot wise in the spatial RNA validation cohort (n = 5). **g**, Representative immunofluorescence staining showing co-localization of CD68 (orange), SELENOP (green), CD8A (red), PD-1 (magenta) and DAPI (blue) in HGSOC samples. Scale bars of each group, 100 μm (left) and 50 μm (right). The yellow arrow points to the CD68$^+$SELENOP$^+$ TAM and the white arrow points to the CD8$^+$PD-1$^+$ T cell. TAM, tumor-associated macrophages. **h,** Scatterplot showing the Spearman correlation between antigen processing and presentation signature scores of *SELENOP*$^+$ macrophages and *GZMB* expression (left) and cytotoxic score (right) of T04_CD8T-GZMH. Colored by samples. For **a-c**, total n = 34 scRNA-seq cohort samples, biological replicates. For **h**, n = 17 solid site samples, biological replicates. For **b**, **f, h**, *P*-value are calculated by the two-sided Spearman correlation test with Benjamini-Hochberg adjustment. Source data are provided as a Source Data file.

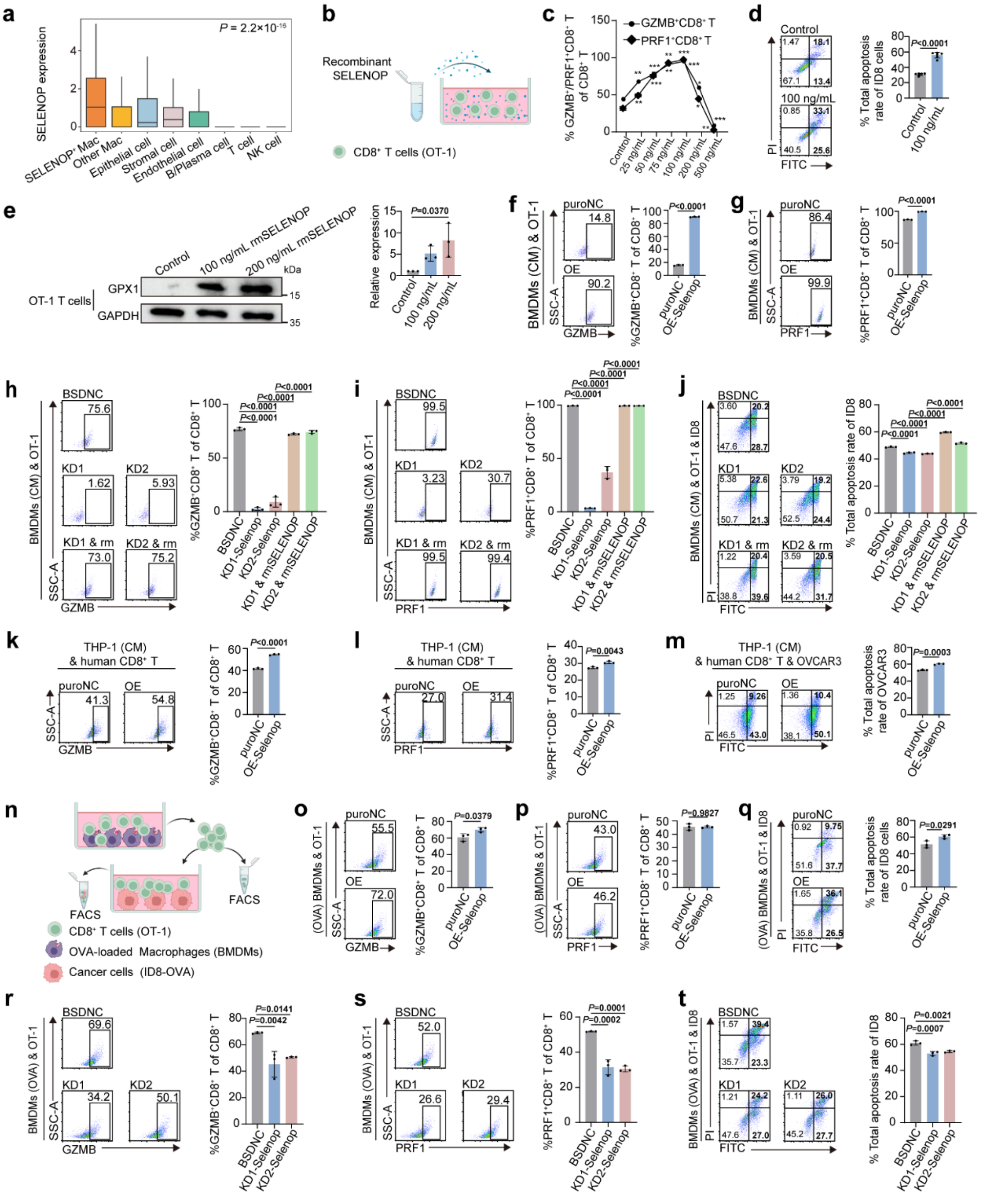

Supplementary Fig. 4 The functional impact of macrophage-derived SELENOP on CD8$^+$ T cells. a, Box plots showing the SELENOP expression across different cell types. n = all 34 scRNA-seq cohort samples, biological replicates. Box represents median ± interquartile range, the whiskers extend up to the maximum values. Two sided Kruskal−Wallis test with Bonferroni post hoc test. **b,** CD8$^+$ T cell cytotoxicity followed by recombinant SELENOP treatment. Created in BioRender. Song, X. (2025) https://BioRender.com/uy61xkt. **c,** The proportions of CD3$^+$CD8$^+$GZMB$^+$ or PRF1$^+$ T cells of OVA-specific OT-1 T cells followed by rm SELENOP treatment or PBS as control. *P* value for CD3$^+$CD8$^+$GZMB$^+$ cells (upper line) are 0.001, 0.0002, 0.0016, 0.0007, 0.0116, 0.0004, respectively (from control to 500 ng/mL). *P* value for CD3$^+$CD8$^+$PRF1$^+$ cells (upper line) are 0.0039, 0.0002, 0.0015, 0.0009, 0.0358, 0.0068, respectively (from control to 500 ng/mL). rmSELENOP, recombinant mouse SELENOP. $^*P < 0.05$, $^{**}P < 0.01$, $^{***}P < 0.001$. **d,** ID8-OVA cells co-cultured

with OVA-specific OT-1 T cells following treated with rm SELENOP or PBS as control. The proportions of apoptosis of ID8-OVA cells. n = 6, biological replicates. **e**, Western blot images and quantifications of GPX1 in OT-1 T cells following treated with rmSELENOP or PBS as control. The proportions of $CD3^+CD8^+GZMB^+$ or $PRF1^+$ T cells of OT-1 T cells or human $CD8^+$ T cells co-cultured with CM from BMDMs-puroNC and BMDMs-OE-Selenop (**f, g**); BMDMs-BSDNC, BMDMs-KD1-Selenop, BMDMs-KD2-Selenop, BMDMs-KD1-Selenop and BMDMs-KD2-Selenop supplemented with rmSELENOP (**h, i**); THP-1-puroNC and THP-1-OE-SELENOP (**k, l**), respectively, and the total apoptosis rate of ID8-OVA cells (**j**) or OVCAR3 (**m**) induced by corresponding $CD8^+$ T cells. **n,** Antigen presentation assay. FACS, fluorescence-activated cell sorting. OVA, ovalbumin. Created in BioRender. Song, X. (2025) https://BioRender.com/7mf6d7f. The proportions of $CD3^+CD8^+GZMB^+$ or $PRF1^+$ T cells of OT-1 T cells cocultured directly with BMDMs-puroNC and BMDMs-OE-Selenop (**o, p**); BMDMs-BSDNC, BMDMs-KD1-Selenop, BMDMs-KD2-Selenop, BMDMs-KD1-Selenop and BMDMs-KD2-Selenop supplemented with rmSELENOP (**r, s**), respectively, and the total apoptosis rate of ID8-OVA cells (**q, t**) induced by corresponding $CD8^+$ T cells. For, **f-j**, **o-t**, BMDMs, bone marrow derived macrophages; puroNC, BMDMs transfected with negative control lentivirus; OE-Selenop, BMDMs overexpressing *Selenop* after lentiviral transfection. BSDNC, BMDMs transfected with negative control shRNA; KD1/KD2-Selenop, BMDMs with *Selenop* knockdown after transfected with sh*Selenop*-1 or sh*Selenop*-2, respectively. For, k-m, puroNC, THP-1 transfected with control negative lentivirus; OE-SELENOP, THP-1 overexpressing *SELENOP* after lentiviral transfection. For **c-m, o-t**, data represent the mean ± SD. For **d-m, o-t**, n = 3 except for **d**, biological replicates. One-way ANOVA with Bonferroni post hoc test for multiple groups, two-sided unpaired Student's t test for two groups, For **c**, n = 3, biological replicates. Brown-Forsythe and Welch ANOVA test. Source data are provided as a Source Data file.

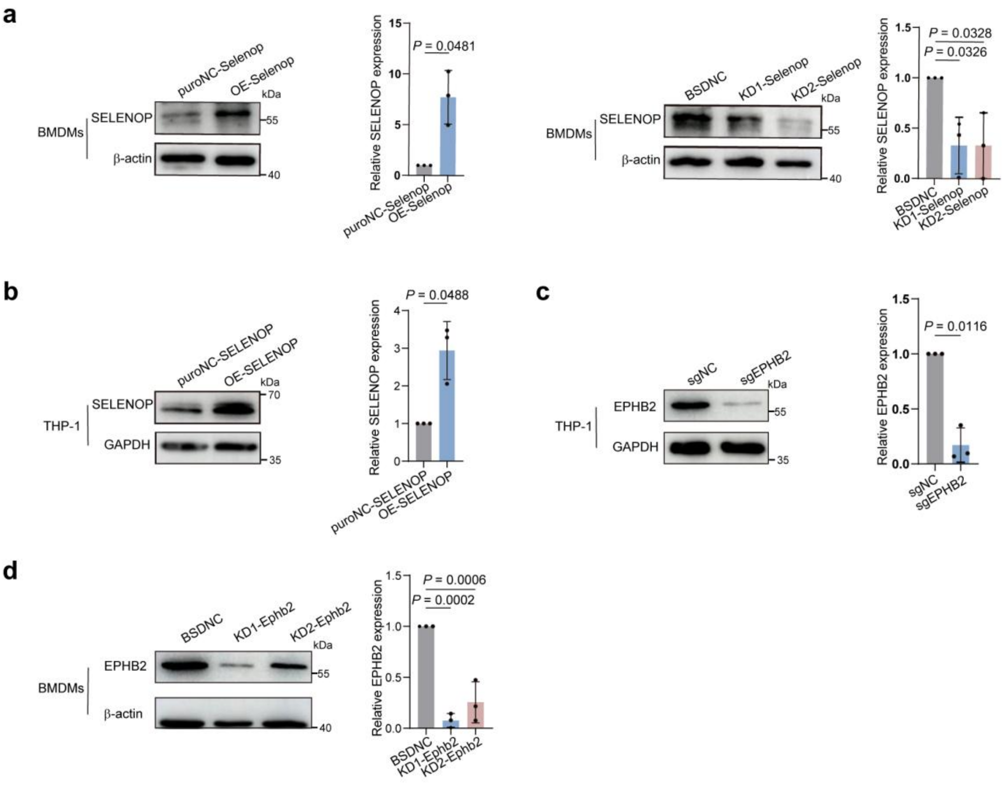

Supplementary Fig. 5 Construction and validation of cell models. a, Quantification of SELENOP by Western blot in BMDMs after infected with a lentivirus encoding *Selenop* (**left**), or after *Selenop* knockdown by sh*Selenop*-1 (KD1) or sh*Selenop*-2 (KD2) (**right**). **b,** Quantification of SELENOP by Western blot in THP-1 after infected with a lentivirus encoding *SELENOP*. **c,** Western blot analysis confirmed CRISPR/Cas9-mediated EPHB2 deletion in THP-1 cells. **d,** Quantification of EPHB2 by Western blot in BMDMs after *Ephb2* knockdown by sh*Ephb2*-1 (KD1) or sh *Ephb2*-2 (KD2). For **a**, **b**, the abbreviations are the same as Supplementary Fig. 4. For **a**, **b**, the abbreviations are the same as Fig. 6**g**, **i**. Data represent the mean ± SD. Two-sided unpaired Student's t test for two groups, one-way ANOVA with Bonferroni post hoc test for multiple groups, n = 3, biological replicates. Source data are provided as a Source Data file.

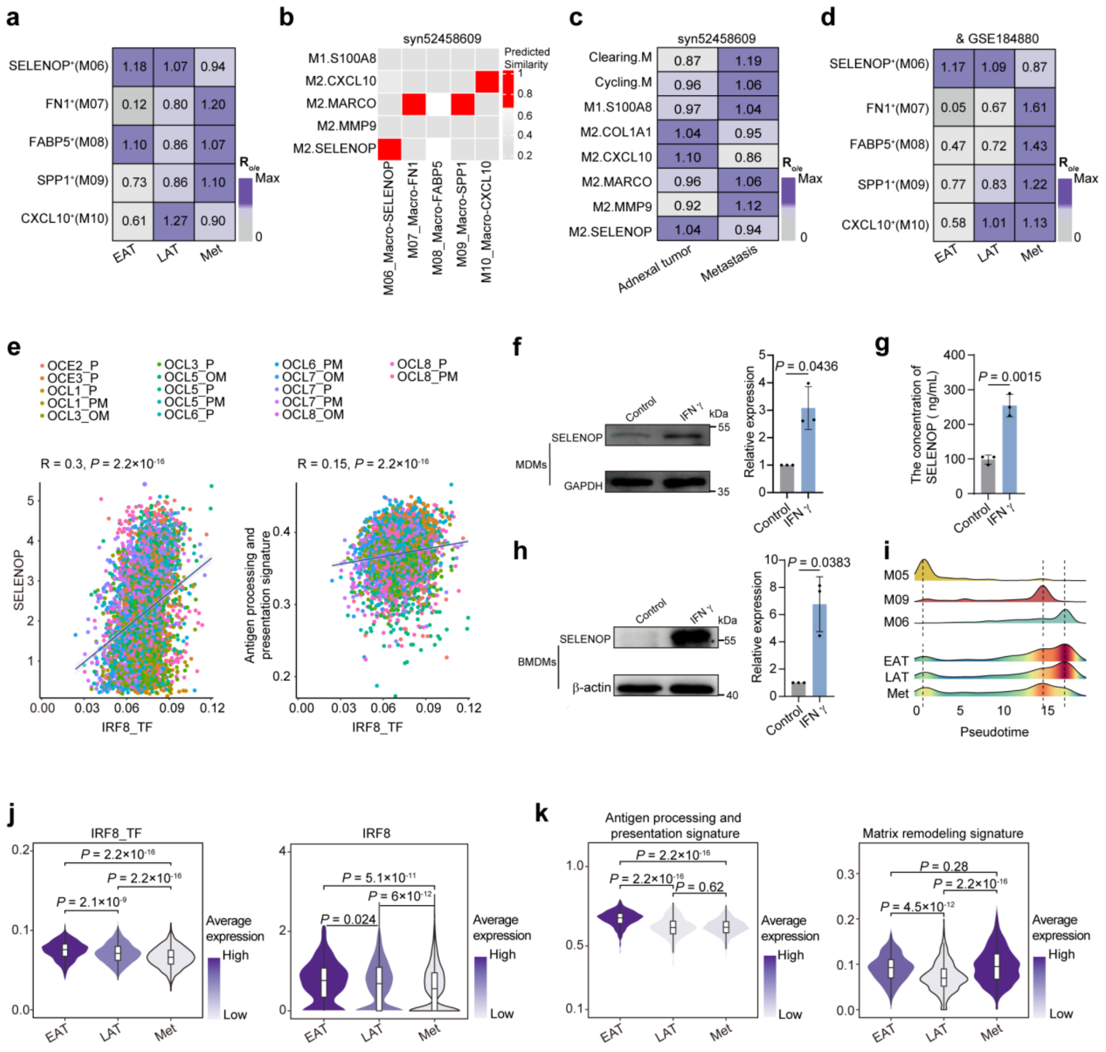

Supplementary Fig. 6 Dynamics of macrophages in the tumor microenvironment associated with HGSOC metastasis. a, Tissue preference of each macrophage clusters across groups, estimated by the $R_{o/e}$ analysis (P = 2.2×10^{-16}). Two-sided chi-squared test. **b,** Heatmaps showing similarity between macrophage subsets identified in our scRNA-seq cohort and those identified in the cohort syn52458609. **c,** Tissue preference of each macrophage clusters in adnexal sites and peritoneal foci in cohort syn52458609, estimated by the $R_{o/e}$ analysis (P = 2.2×10^{-16}). Two-sided Chi-squared test. **d,** Tissue preference of each macrophage clusters estimated by $R_{o/e}$ (integrated GSE184880 with our scRNA-seq data). (P = 2.2×10^{-16}). Two-sided Chi-squared test. **e,** Scatterplots showing the Spearman correlations between IRF8 transcription factor (TF) activity and *SELENOP* expression and antigen processing and presentation signature score of *SELENOP*$^{+}$ macrophages. Two-sided Spearman correlation test with Benjamini-Hochberg adjustment. Western blot analysis and quantification of SELENOP expression in MDMs (**f**) or BMDMs (**h**) after treatment with PBS as control or IFNγ. **g**, ELISA showing SELENOP level in the corresponding culture supernatants in **f**. **i,** Density plot showing the density patterns of cells from different macrophage clusters (upper) and cells from different solid sites (lower) along the pseudotime. **j,** Violin plots showing IRF8 activity (left) and expression (right) of *SELENOP*$^{+}$ macrophages across groups. **k,** Violin plots showing antigen processing and presentation signature scores of *SELENOP*$^{+}$ macrophages (left) and

matrix remodeling signature scores of *SPP1*$^+$ macrophages (right) across groups. For **j, k,** Two-sided Wilcoxon tests, adjusted by the Benjamini-Hochberg procedure. Colored by average expression. For **a, e, i, j, k**, n = 17 scRNA-seq cohort solid site samples, biological replicates. For **d**, total of n = 24 samples, n = 7 EAT, n = 8 LAT, n = 9 Met, biological replicates. For **f**, **g**, **h**, data represent the mean ± SD. For **j, k,** box represents median ± interquartile range, and the whiskers extend up to the minimum and maximum values. For **f-h**, two-sided unpaired Student's t test, n = 3, biological replicates. Source data are provided as a Source Data file.

a

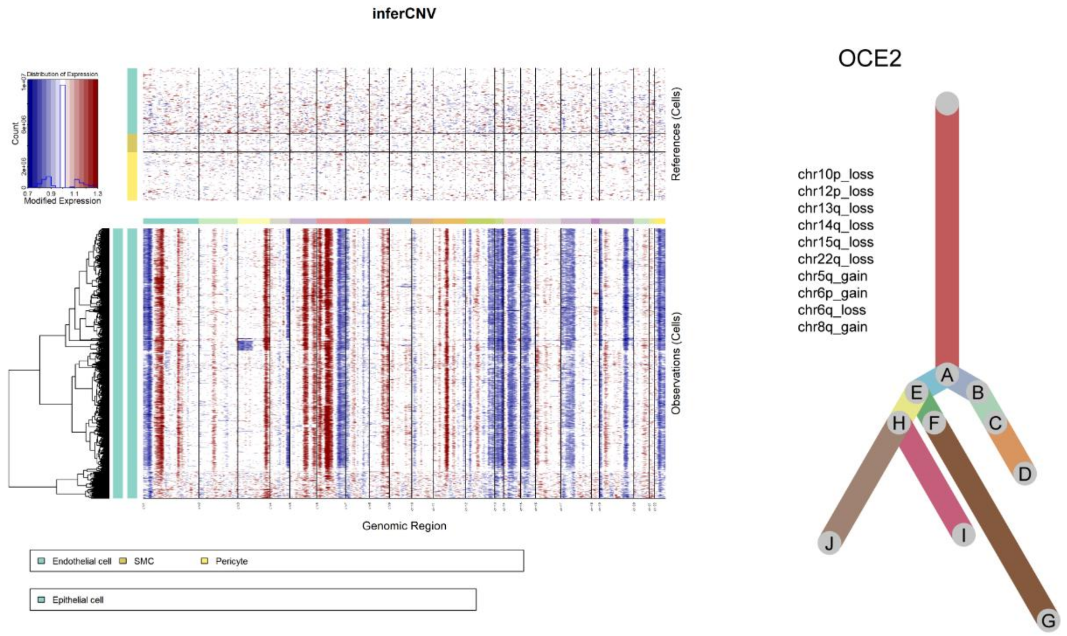

b

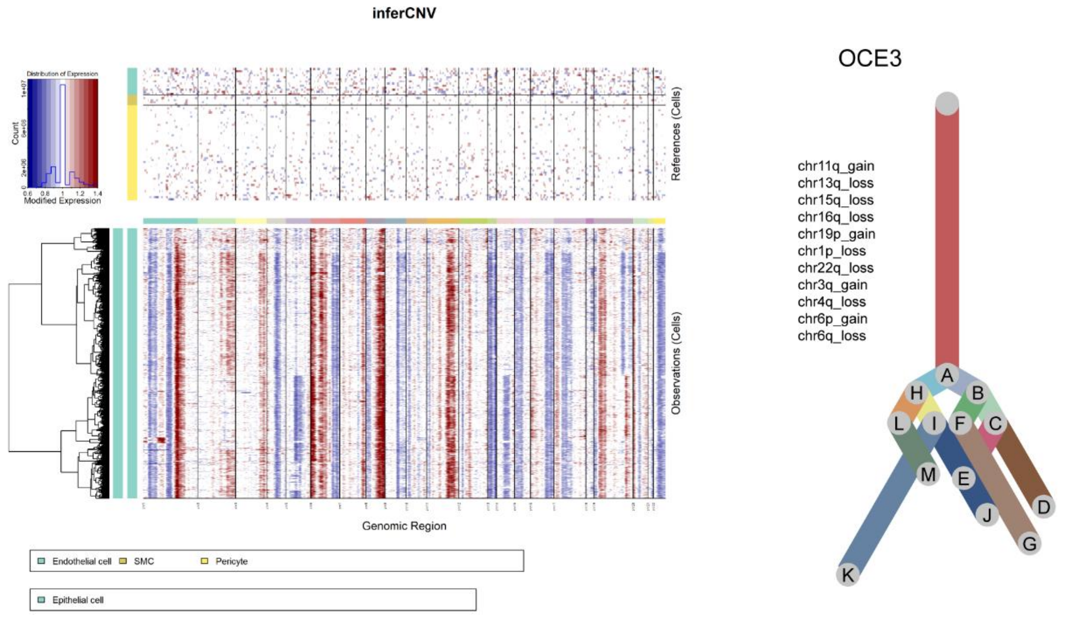

Supplementary Fig. 7 Malignant epithelial cell genomic alterations in patients OCE2 and OCE3. Copy number heatmap indicating chromosomal copy number variation (CNV) of endothelial cells, SMC cells, pericyte cells and malignant epithelial cells of each HGSOC patient. Upper panel shows the CNVs of endothelial cells, SMC cells, and pericyte cells as reference cells. Lower panel shows the CNVs of malignant epithelial cells from patient OCE2 (**a**, left) AND (**b**, left) OCE3 for observation. Each row of the heatmap represents a single cell. Deletions (blue) or amplifications (red) were inferred by averaging expression on the respective chromosomes. Gains or losses were inferred by averaging expression over 100 gene stretch on the respective chromosomes. The evolutionary phylogenetic trees of malignant cells from patients OCE2 (**a**, right) and OCE3 (**b**, right). The length of each branch is proportional to the number of cells in each subclone containing the corresponding CNVs. Some key CNV events were labeled in the clonality tree.

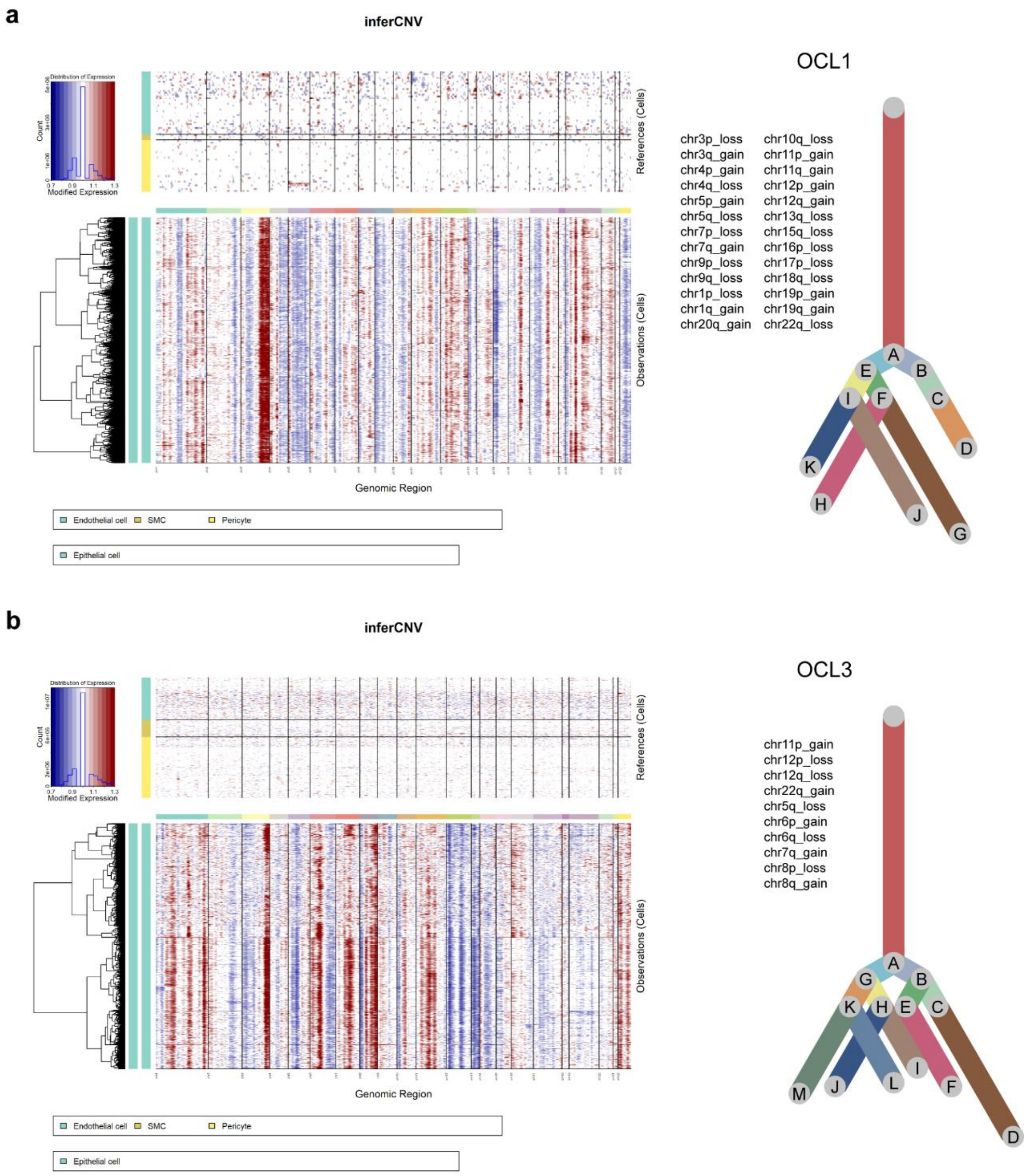

Supplementary Fig. 8 Malignant epithelial cell genomic alterations in patients OCL1 and OCL3. Copy number heatmap indicating chromosomal copy number variation (CNV) of endothelial cells, SMC cells, pericyte cells and malignant epithelial cells of each HGSOC patient. Upper panel shows the CNVs of endothelial cells, SMC cells, and pericyte cells as reference cells. Lower panel shows the CNVs of malignant epithelial cells from patient OCL1 (**a**, left) AND OCL3 (**b**, left) for observation. Each row of the heatmap represents a single cell. Deletions (blue) or amplifications (red) were inferred by averaging expression on the respective chromosomes. Gains or losses were inferred by averaging expression over 100 gene stretch on the respective chromosomes. The evolutionary phylogenetic trees of malignant cells from patients OCL1 (**a**, right) and OCL3 (**b**, right). The length of each branch is proportional to the number of cells in each subclone containing the corresponding CNVs. Some key CNV events were labeled in the clonality tree.

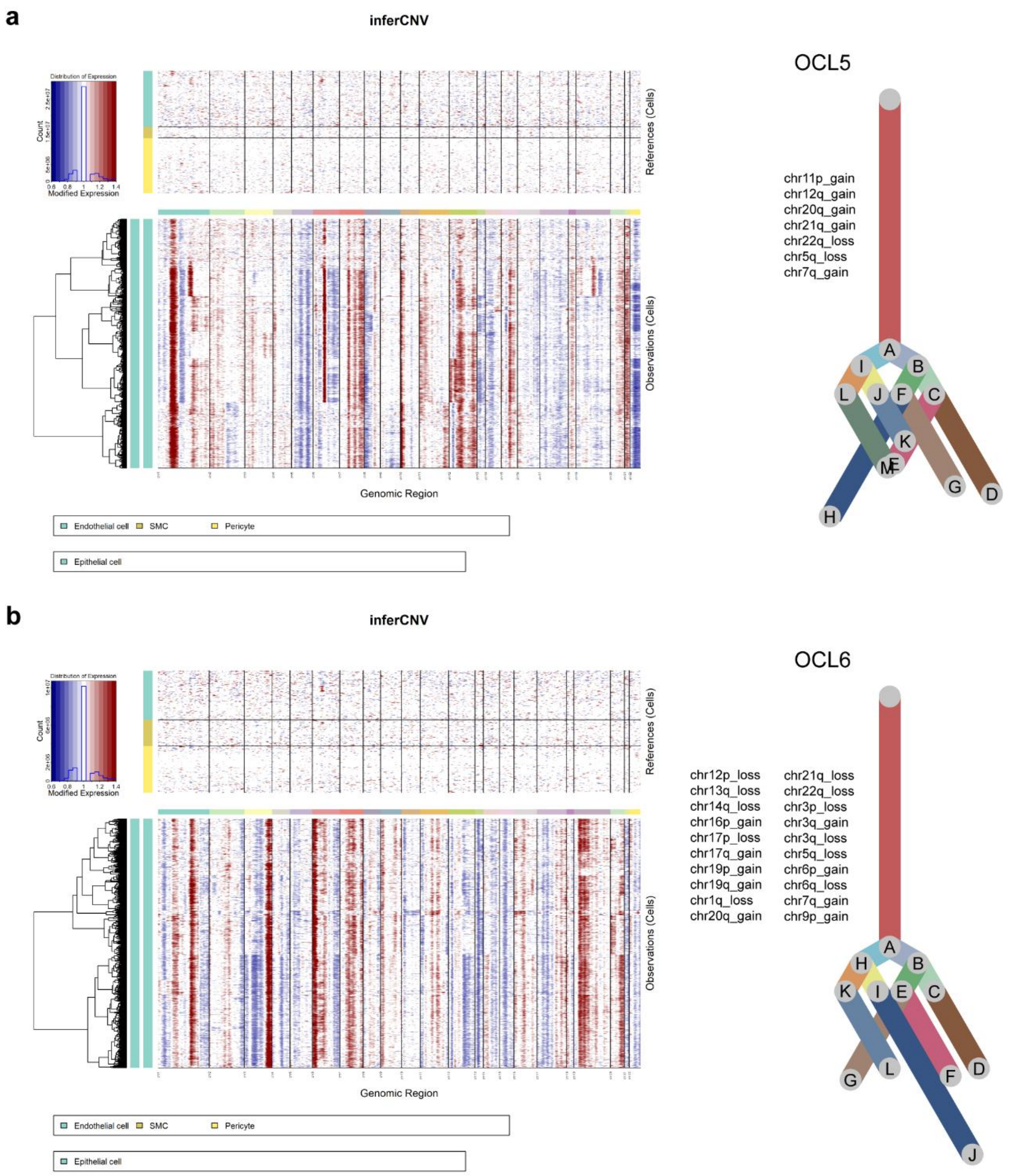

Supplementary Fig. 9 Malignant epithelial cell genomic alterations in patients OCL5 and OCL6. Copy number heatmap indicating chromosomal copy number variation (CNV) of endothelial cells, SMC cells, pericyte cells and malignant epithelial cells of each HGSOC patient. Upper panel shows the CNVs of endothelial cells, SMC cells, and pericyte cells as reference cells. Lower panel shows the CNVs of malignant epithelial cells from patient OCL5 (**a**, left) AND OCL6 (**b**, left) for observation. Each row of the heatmap represents a single cell. Deletions (blue) or amplifications (red) were inferred by averaging expression on the respective chromosomes. Gains or losses were inferred by averaging expression over 100 gene stretch on the respective chromosomes. The evolutionary phylogenetic trees of malignant cells from patients OCL5 (**a**, right) and OCL6 (**b**, right). The length of each branch is proportional to the number of cells in each subclone containing the corresponding CNVs. Some key CNV events were labeled in the clonality tree.

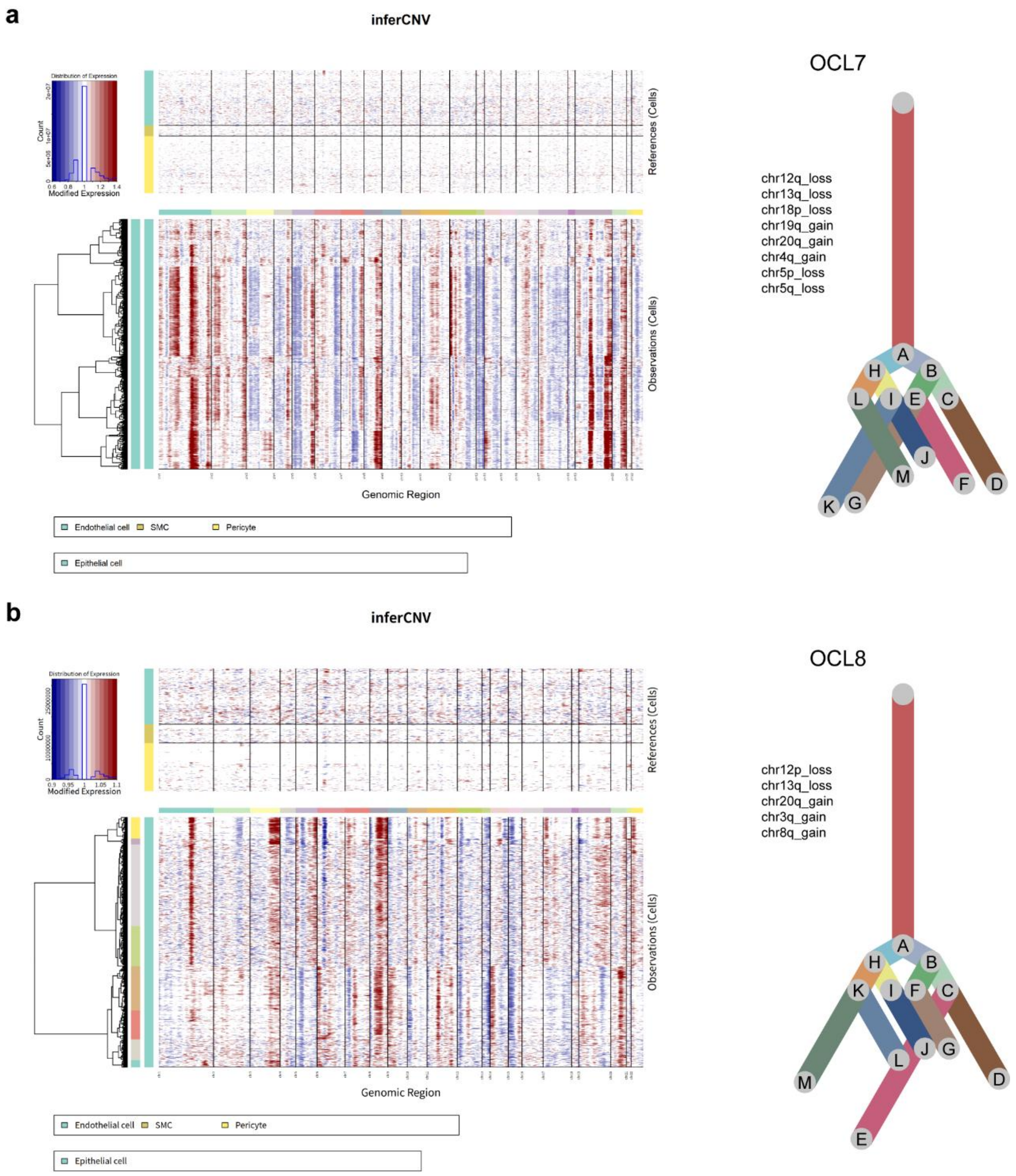

Supplementary Fig. 10 Malignant epithelial cell genomic alterations in patients OCL7 and OCL8. Copy number heatmap indicating chromosomal copy number variation (CNV) of endothelial cells, SMC cells, pericyte cells and malignant epithelial cells of each HGSOC patient. Upper panel shows the CNVs of endothelial cells, SMC cells, and pericyte cells as reference cells. Lower panel shows the CNVs of malignant epithelial cells from patient OCL7 (**a**, left) AND OCL8 (**b**, left) for observation. Each row of the heatmap represents a single cell. Deletions (blue) or amplifications (red) were inferred by averaging expression on the respective chromosomes. Gains or losses were inferred by averaging expression over 100 gene stretch on the respective chromosomes. The evolutionary phylogenetic trees of malignant cells from patients OCL7 (**a**, right) and OCL8 (**b,** right). The length of each branch is proportional to the number of cells in each subclone containing the corresponding CNVs. Some key CNV events were labeled in the clonality tree.

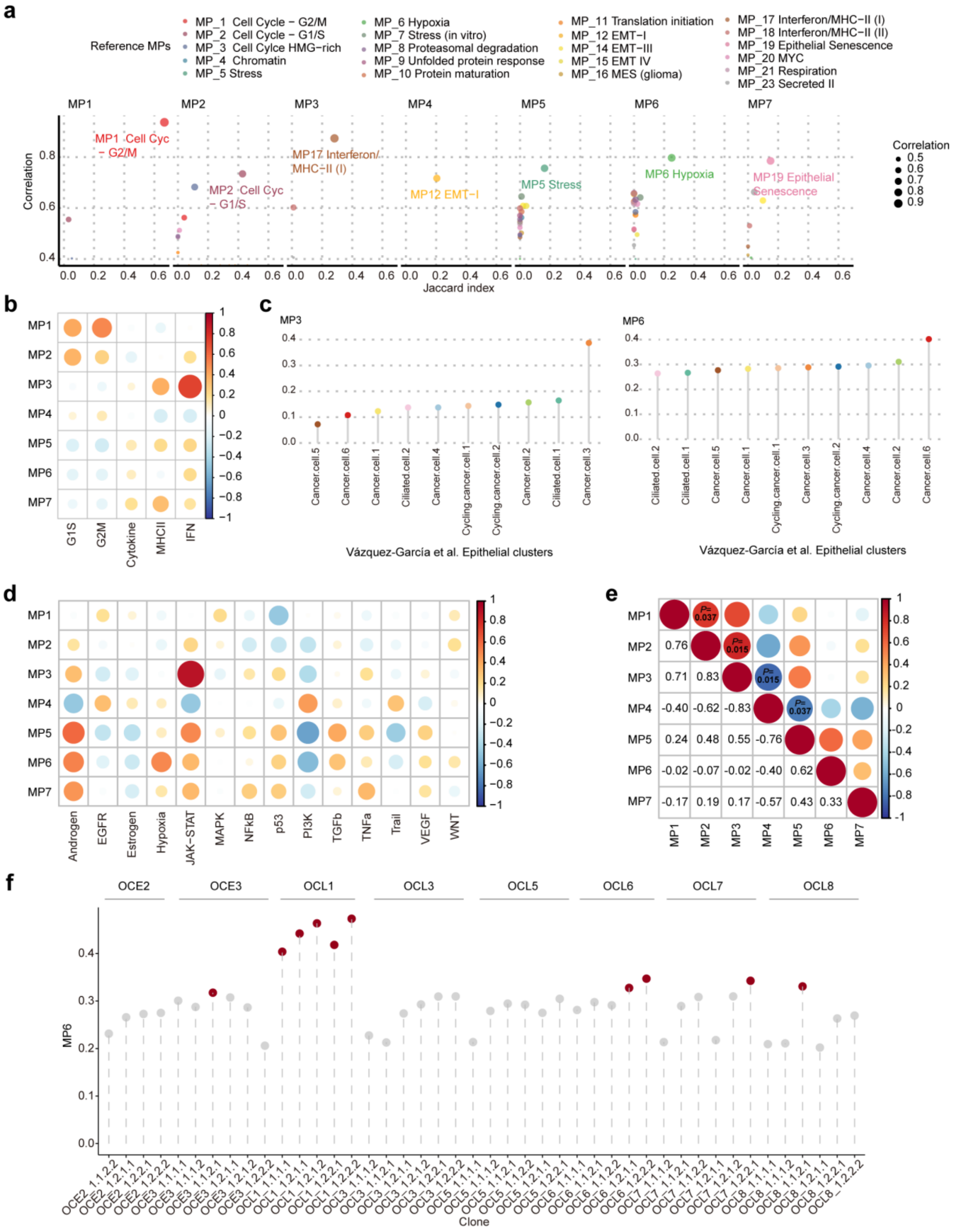

Supplementary Fig. 11 Dissection of the characteristics of malignant epithelial cells in the tumor microenvironment associated with HGSOC metastasis. a, Scatter plot characterizing MPs by Jaccard Index (x-axis) and the Spearman correlation between MPs signature score in our data and MPs signature score in an independent cohort in tumor cells (y-axis) from Gavish, A. *et al* (ref. 55). **b,** A Spearman correlation dot plot showing the MPs signature score in our data and the MPs signature score in an independent cohort of malignant epithelial cells from Izar, B. *et al* (ref. 56). Dot size and color represent correlation coefficients. **c,** Lollipop plot showing Spearman correlation between MP3 (left) and MP6 (right) signature scores in our data and different malignant epithelial cell clusters in an independent cohort syn52458609. The color of the circles represents different cell

populations. **d,** A Spearman correlation dot plot between MPs signature score in our data and PROGENy pathway activity score. Dot size and color represent correlation coefficients. **e**, Heatmap displays the Spearman correlation coefficients calculated among the signature scores of distinct malignant epithelial cells MPs of this study. *P*-values are calculated by Spearman correlation test. **f**, Lollipop plot showing the MP6 signature scores of subclones in each patient, and subclones with top ten MP6 signature score were labeled by red color. For **a-f**, data were summarized from all n = 21 scRNA-seq cohort samples, including n = 17 solid site samples of HGSOC and n = 4 Ascites (from OCL3, OCL5, OCL7 and OCL8), biological replicates. *P*-value are calculated by the two-sided Spearman correlation test with Benjamini-Hochberg adjustment.

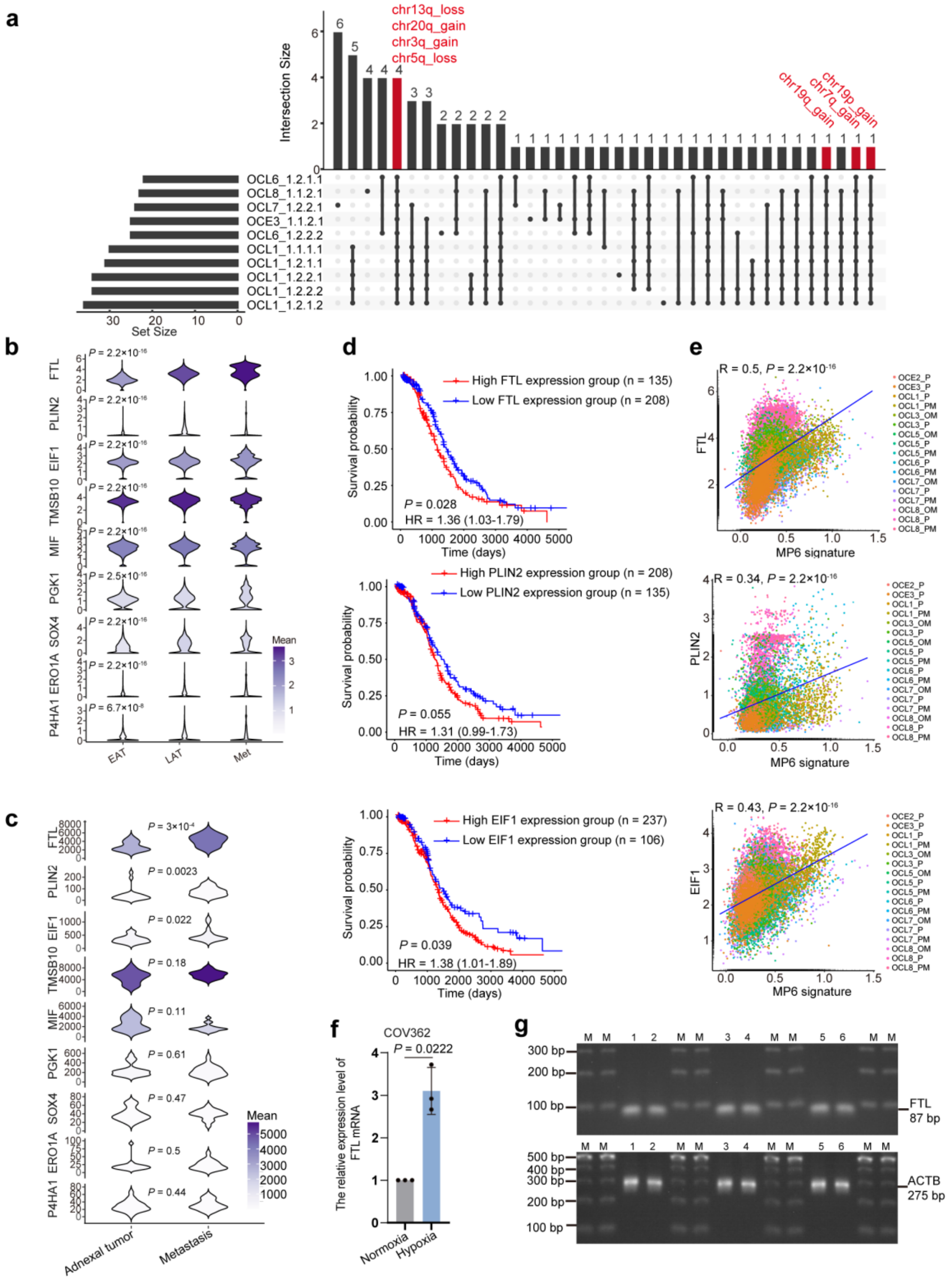

Supplementary Fig. 12 Dissection of the dynamics of tumor cells in the tumor microenvironment associated with HGSOC metastasis. a, UpSet plots revealed the numbers of chromosomes shared by the subclones with the top ten MP6 signature score among patients. The red bars and dots represent the chromosomes shared by ten or nine subclones. Their chromosomal variations are annotated above the bar chart. **b,** Violin plots displaying the expression levels of nine up regulated genes (*FLT, PLIN2, EIF1, TMSB10, MIF, PGK1, SOX4, ERO1A, P4HA1*) from the top 50 MP6-scoring genes across the EAT, LAT, and Met groups. *P*-values are calculated by the two side Kruskal−Wallis test with Bonferroni post hoc test. and are colored by average expression. **c,** Violin plots displaying the expression levels of *FLT, PLIN2, EIF1, TMSB10, MIF, PGK1, SOX4, ERO1A,* and *P4HA1* in adnexal tumors (n = 20) and metastasis (n = 18) in the bulk RNA-seq cohort

from Yang *et al.* (ref. 15). *P* values are calculated by two-sided Wilcoxon test. Colored by average expression. **d,** The Kaplan-Meier overall survival curves of patients with HGSOC grouped based on the expression of the *FTL* (upper), *PLIN2* (middle), and *EIF1* (lower). *P*-values were determined by log-rank test (n = 343). **e,** Scatter plots showing the Spearman correlations between *FTL* (upper), *PLIN2* (middle), and *EIF1* (lower) and MP6 signature scores in malignant epithelial cells, colored by different samples. *P* value is calculated by the two-sided Spearman correlation test with Benjamini-Hochberg adjustment. **f,** Real-time PCR analysis of the levels of *FTL* mRNA in COV362 cell line cultured under normoxic or hypoxic conditions. Data represent the mean ± SD. *P*-values are calculated by two-sided unpaired Student's t test. n = 3, biological replicates. **g,** Size and purity determination of amplified *FTL* (upper) and *ACTB* (lower) on 1.5% TBE agarose gels. Line 1, 3, 5: amplified products of RT-qPCR from COV362 cell line cultured under normoxic condition. Line 2, 4, 6: amplified products of RT-qPCR from COV362 cell line cultured under hypoxic condition. M: marker. For **a,** data were summarized from all n = 21 HGSOC samples, including n = 17 solid sites samples of HGSOC and n = 4 Ascites (from OCL3, OCL5, OCL7 and OCL8), biological replicates. For **b, e**, all n = 17 solid sites samples were analyzed, biological replicates. Source data are provided as a Source Data file.

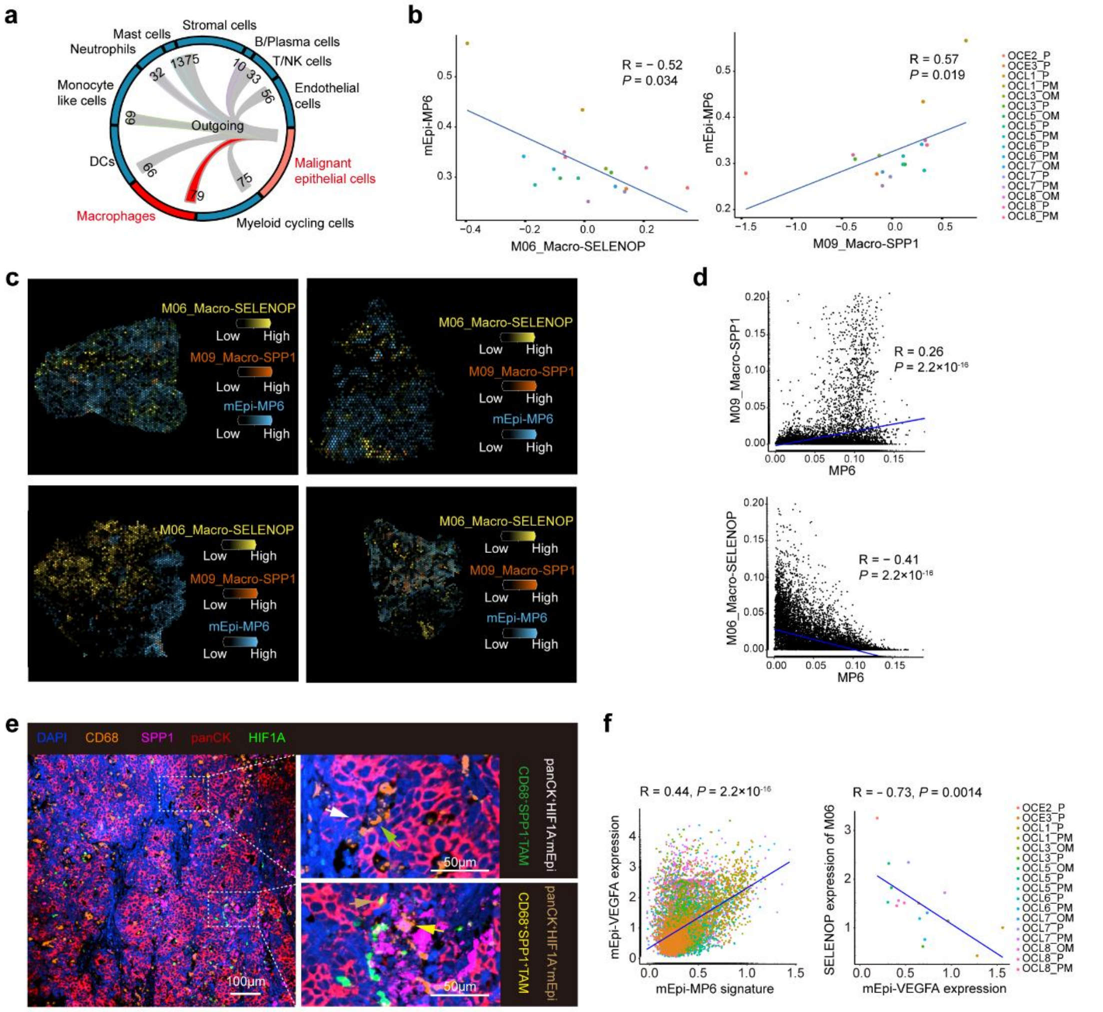

Supplementary Fig. 13 Dissection of the relationship between hypoxia-driven malignant epithelial cells and macrophages enriched in the solid tumor microenvironment of HGSOC. a, Chord diagrams showing links between predicted ligands from malignant epithelial cells with their associated receptors found on other type cells in solid sites. The interaction numbers of the ligand-receptor interaction were labeled. **b,** Scatterplots showing the Spearman correlations between MP6 signature scores of malignant epithelial cells and the proportion of *SELENOP*$^{+}$ macrophages (left) and *SPP1*$^{+}$ macrophages (right). **c** Representative spatial co-localizations of *SPP1*$^{+}$ macrophage with malignant epithelial cells with MP6 signature in the special RNA validation cohort (n = 5). **d,** Scatterplot showing the Pearson correlation between MP6 signature scores of malignant epithelial cells and *SPP1*$^{+}$ macrophage (upper) or *SELENOP*$^{+}$ macrophages (lower) abundance. **e,** Representative immunofluorescence staining showing co-localization of CD68 (orange), SPP1 (magenta), panCK (red), HIF1A (green) and DAPI (blue) in HGSOC samples. Scale bars of each group, 100 μm (left) and 50 μm (right). The white arrow points to the panCK^{+}HIF1A^{-} cell, the green arrow point to the CD68^{+}SPP1^{-} cell, the brown arrow points to the panCK^{+}HIF1A^{+} cell, and the yellow arrow points to the CD68^{+}SPP1^{+} cell. **f,** Scatterplots showing the Spearman correlations between MP6 signature and *VEGFA* expression of the malignant epithelial cells (left); *VEGFA*

expression of the malignant epithelial cells and the expression of *SELENOP* in *SELENOP*$^{+}$ macrophages (M06) (right), colored by different samples. For **a, b, f,** n = 17 scRNA-seq cohort solid site samples, biological replicates. For **b**, **d**, **f**, *P*-value are calculated by the two-sided Spearman correlation test with Benjamini-Hochberg adjustment.

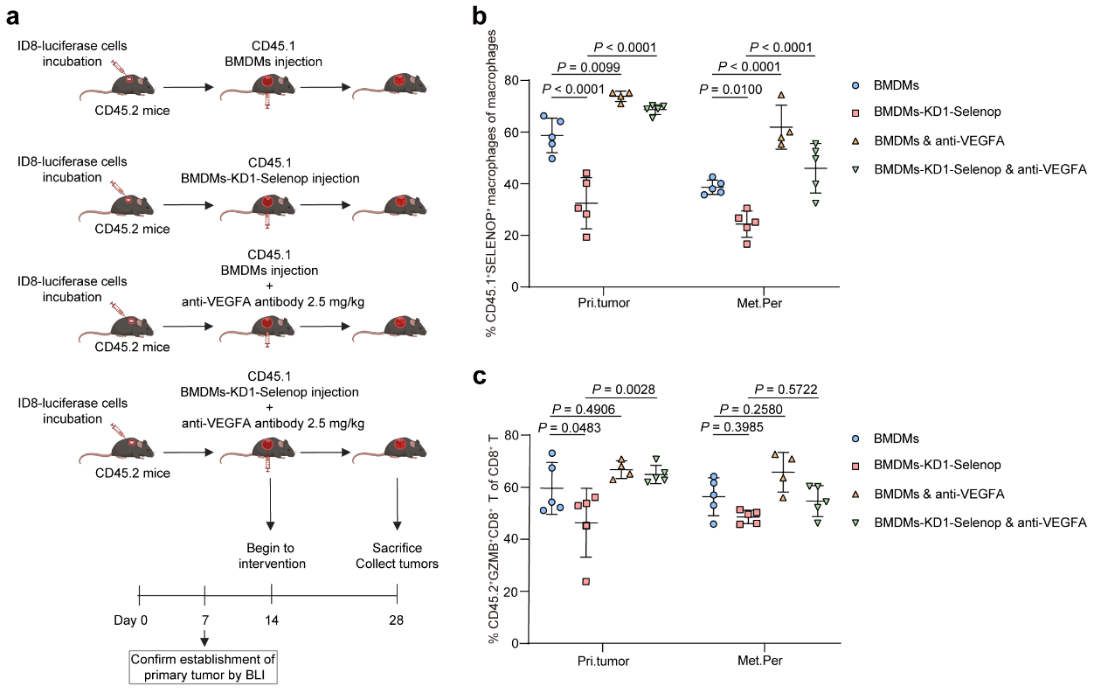

Supplementary Fig. 14 Exploration of the capacity of SELENOP$^+$ macrophages to directly prime CD8$^+$ T cell and involvement in VEGFA within ovarian tumor microenvironment *in vivo.*

a, Schematic diagram of the protocol for adoptive cellular transfer experiments. Fresh primary tumor tissues were digested and stained for flow cytometry analysis. Created in BioRender. Song, X. (2025) https://BioRender.com/xqsyso9. Shown is the proportion of CD45.1$^+$F4/80$^+$CD11b$^+$SELENOP$^+$ cells (**b**) and CD45.2$^+$CD3$^+$CD8$^+$GZMB$^+$ cells (**c**) across different sites of each group. Pri. tumor, primary tumor of orthotopic ovarian cancer mouse model; Met.Per, peritoneal metastasis of orthotopic ovarian cancer mouse model. n = 5 in BMDMs group, n = 5 in BMDMs-KD1-Selenop group, n = 4 in BMDMs combined with anti-VEGFA group, n = 5 in BMDMs-KD1-Selenop combined with anti-VEGFA group, biological replicates. Data represent the mean ± SD. *P*-values are calculated by two-way ANOVA with Tukey post hoc test. Source data are provided as a Source Data file.

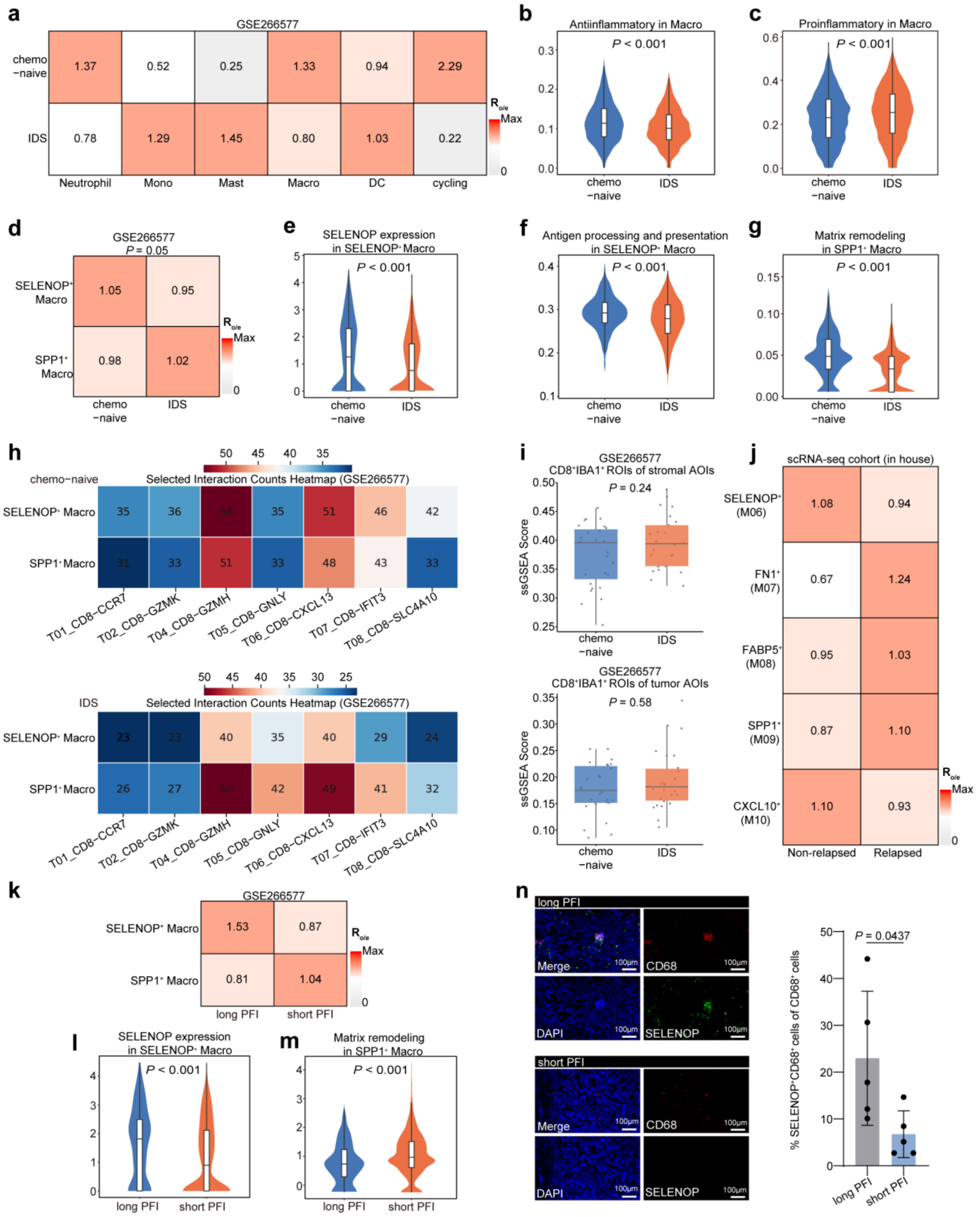

Supplementary Fig. 15 Reduced interaction potential between *SELENOP*$^{+}$ Macrophages and *GZMH*$^{+}$ precursor exhausted CD8^{+} T Cells following chemotherapy. a, Tissue preference of myeloid clusters estimated by $R_{o/e}$ between chemo-naive and post-neoadjuvant chemotherapy (interval debulking surgery, IDS) groups in GSE266577 ($P = 2.2\times10^{-16}$). Violin plots comparing the proinflammatory (**b**) and anti-inflammatory function scores (**c**) between groups. **d,** Tissue preference of inferred *SELENOP*$^{+}$ and *SPP1*$^{+}$ macrophages estimated by $R_{o/e}$ between groups in GSE266577. Violin plots comparing the *SELENOP* expression (**e**), antigen processing and presentation of *SELENOP*$^{+}$ macrophages (**f**) and matrix remodeling signature scores of *SPP1*$^{+}$ macrophages (**g**) between groups. **h,** Heatmaps illustrating the cell-cell interaction patterns in

chemo-naive (upper) and IDS samples (lower) groups. **i,** Boxplots showing the coexistence score in stromal (upper) or tumor (lower) areas of interest of *SELENOP*$^+$ macrophages and *GZMH*$^+$ precursor exhausted CD8$^+$ T cells between groups. AOIs, areas of interest. **j,** Tissue preference of *SELENOP*$^+$ and *SPP1*$^+$ macrophages estimated by $R_{o/e}$ between groups in our scRNA-seq dataset (P = 2.2×10^{-16}). **k,** Tissue preference of inferred *SELENOP*$^+$ and *SPP1*$^+$ macrophages estimated by $R_{o/e}$ between short and long platinum free interval (PFI) groups in GSE266577 (P = 2.2×10^{-16}). Violin plots comparing the *SELENOP* expression of *SELENOP*$^+$ macrophages (**l**), and matrix remodeling signature scores of *SPP1*$^+$ macrophages (**m**) between groups. **n,** Representative image of ovarian tumor stained by multiplex immunohistochemistry (left), scale bar, 100 μm and the quantification plots (right). Data represent the mean ± SD. Two-sided unpaired Student's t test, n =10, biological replicates. For **a-g**, a total of n = 18 HGSOC paired samples, including n = 9 chemo-naive, n= 9 IDS, biological replicates. For **h**, n = 26 chemo-naive, n= 20 IDS, biological replicates. For **i** (upper), n = 27 chemo-naive, n= 25 IDS, biological replicates. For **i** (lower), n = 26 chemo-naive, n= 26 IDS, biological replicates. For **j**, n = 7 solid sites samples in non-replased group, n= 8 solid sites samples in replsed group, biological replicates. For **k-m**, a total of n = 22 HGSOC samples, including n = 6 long PFI, n= 16 short PFI, biological replicates. For **a, d, j, k,** *P*-values: two-sided chi-squared test. For **b, c, e-g, i, l, m**, box, median ± interquartile range, and the whiskers extend up to the minimum and maximum values. *P*-values: two-sided Wilcoxon test. Source data are provided as a Source Data file.

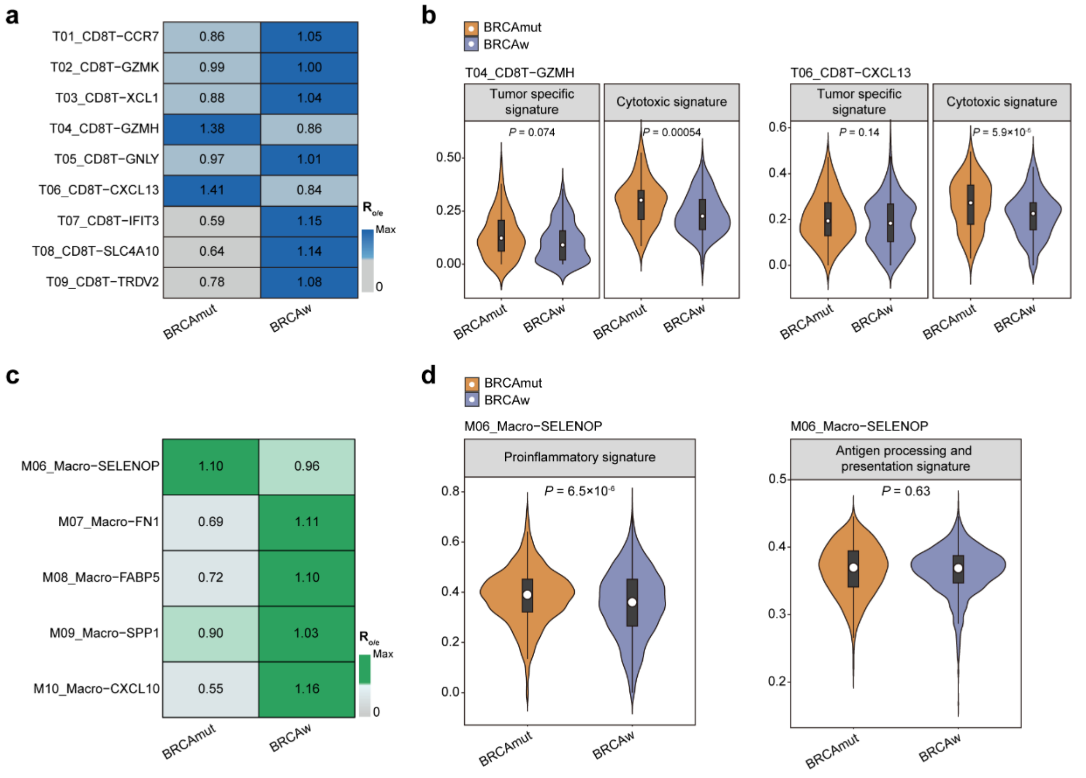

Supplementary Fig. 16 Distinct immune cell subpopulations enriched in solid sites across different *BRCA* statuses. a, Tissue preference of conventional $CD8^+$ T subpopulation between BRCAmut and BRCAw groups, estimated by $R_{o/e}$ ($P = 9.725\times10^{-10}$). P-values are calculated by two-sided chi-squared test. **b,** Violin plots showing tumor specific and cytotoxic signature scores of T04_CD8T-GZMH (left) and T06_CD8T-CXCL13 (right) between BRCAmut and BRCAw groups. P values calculated by two-sided Wilcoxon tests. BRCAmut, *BRCA* mutant group. BRCAw, *BRCA* wild-type group. **c,** Tissue preference of macrophage subpopulation between BRCAmut and BRCAw groups, estimated by $R_{o/e}$ ($P = 0.00000971$). P-values are calculated by two-sided chi-squared test. **d**, Violin plots showing proinflammatory (left) and antigen processing and presentation (right) signature scores of *SELENOP*$^+$ macrophages between BRCAmut and BRCAw groups. P values calculated by two-sided Wilcoxon tests. For **a-d**, data were summarized from all n = 6 samples. BRCAw: n = 4, BRCAmut: n = 2, biological replicates. For **b, d**, Box of violin plot represents median± interquartile range, and the whiskers extend up to the minimum and maximum values. Source data are provided as a Source Data file.

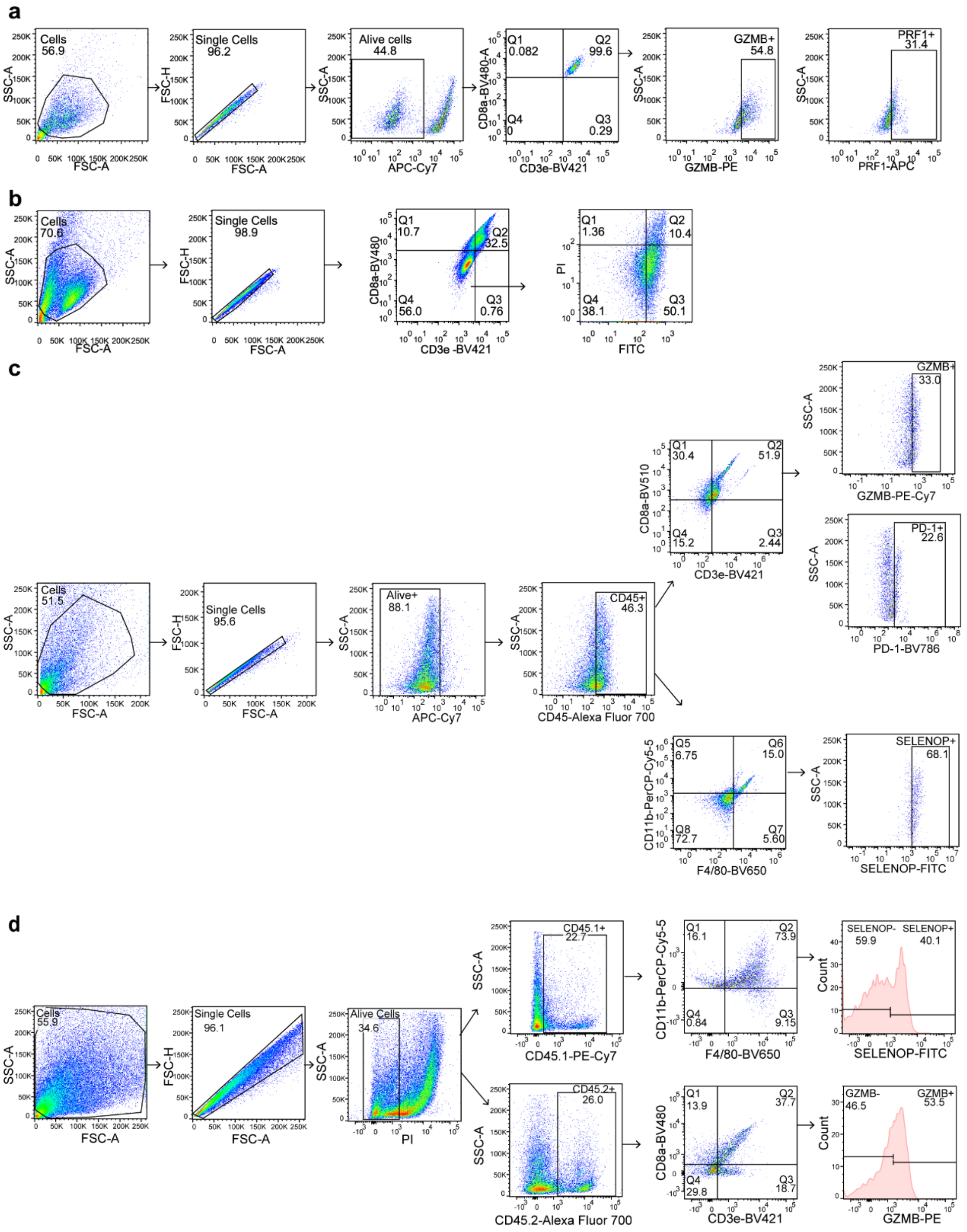

Supplementary Fig. 17 Representative gating strategy. a, GZMB^{+} CD8^{+} T cells (CD3e+CD8a+GZMB+), PRF1^{+} CD8^{+} T cells (CD3e+CD8a+PRF1+) and gating strategy for Supplementary Fig. 4c, f-i, k, l, o, p, r, s. **b**, Gating strategy for apoptosis (Apoptosis status was determined by Annexin V-FITC staining. Cell death was determined by propidium iodide (PI) staining. Gating methods for FITC-/PI-(live), FITC+/PI-(early apoptosis), FITC-/PI+ (necrosis), and FITC+/PI+ (late apoptosis) are shown.) for Fig. 3l and Supplementary Fig. 4d, j, m, q, t. **c**, GZMB^{+} CD8^{+} T cells (CD45+CD3e+CD8a+GZMB+), PD-1^{+} CD8^{+} T cells (CD45+CD3e+CD8a+PD-1+) and SELENOP^{+} macrophages gating strategy (CD45+F4/80+CD11b+SELENOP+) for Fig. 7h-j. **d**, CD45.2^{+} GZMB^{+} CD8^{+} T cells

(CD45.2+CD3e+CD8a+GZMB+) and CD45.1^{+} SELENOP^{+} (CD45.1+F4/80+CD11b+SELENOP+) macrophages gating strategy for Supplementary Fig. 14b,c.

Supplementary References

1. Zhang, L., *et al.* Lineage tracking reveals dynamic relationships of T cells in colorectal cancer. *Nature* **564**, 268-272 (2018).
2. Cheng, S., *et al.* A pan-cancer single-cell transcriptional atlas of tumor infiltrating myeloid cells. *Cell* **184**, 792-809.e723 (2021).
3. Yang, Y., *et al.* Pan-cancer single-cell dissection reveals phenotypically distinct B cell subtypes. *Cell* **187**, 4790-4811.e4722 (2024).
4. Zheng, X., *et al.* Single-cell analyses implicate ascites in remodeling the ecosystems of primary and metastatic tumors in ovarian cancer. *Nature cancer* **4**, 1138-1156 (2023).
5. Vázquez-García, I., *et al.* Ovarian cancer mutational processes drive site-specific immune evasion. *Nature* **612**, 778-786 (2022).
6. Yang, B., *et al.* Spatial heterogeneity of infiltrating T cells in high-grade serous ovarian cancer revealed by multi-omics analysis. *Cell reports. Medicine* **3**, 100856 (2022).
7. Pellicci, D.G., Koay, H.F. & Berzins, S.P. Thymic development of unconventional T cells: how NKT cells, MAIT cells and γδ T cells emerge. *Nature reviews. Immunology* **20**, 756-770 (2020).
8. Zhang, L., *et al.* Single-Cell Analyses Inform Mechanisms of Myeloid-Targeted Therapies in Colon Cancer. *Cell* **181**, 442-459.e429 (2020).
9. Azizi, E., *et al.* Single-Cell Map of Diverse Immune Phenotypes in the Breast Tumor Microenvironment. *Cell* **174**, 1293-1308.e1236 (2018).
10. Zhang, Q., *et al.* Landscape and Dynamics of Single Immune Cells in Hepatocellular Carcinoma. *Cell* **179**, 829-845.e820 (2019).
11. Huang, X.Z., *et al.* Single-cell sequencing of ascites fluid illustrates heterogeneity and therapy-induced evolution during gastric cancer peritoneal metastasis. *Nature communications* **14**, 822 (2023).
12. Westphalen, C.B., *et al.* Pan-cancer Analysis of Homologous Recombination Repair-associated Gene Alterations and Genome-wide Loss-of-Heterozygosity Score. *Clinical cancer research : an official journal of the American Association for Cancer Research* **28**, 1412-1421 (2022).
13. Licaj, M., *et al.* Residual ANTXR1+ myofibroblasts after chemotherapy inhibit anti-tumor immunity via YAP1 signaling pathway. *Nature communications* **15**, 1312 (2024).
14. Tanevski, J., Flores, R.O.R., Gabor, A., Schapiro, D. & Saez-Rodriguez, J. Explainable multiview framework for dissecting spatial relationships from highly multiplexed data. *Genome biology* **23**, 97 (2022).
15. Kuppe, C., *et al.* Spatial multi-omic map of human myocardial infarction. *Nature* **608**, 766-777 (2022).
