# Supplementary material for: Hypoxia-driven remodeling of SELENOP+ macrophages shapes T cell dynamics and promotes ovarian cancer metastasis
Source: Nat Commun. 2026 Jan 12;17:1097. doi: 10.1038/s41467-025-67859-2 (PMC12852879; doi:10.1038/s41467-025-67859-2)
Supplement: Supplementary file 2 — Description of Additional Supplementary File [file 41467_2025_67859_MOESM2_ESM.pdf]

Description of Additional Supplementary Files

File Name: Supplementary Data 1
Description: Characteristics of patients with HGSOC and specimens included in our scRNAseq dataset.

File Name: Supplementary Data 2
Description: Raw data of spatial transcriptomics.

File Name: Supplementary Data 3
Description: Major clusters and subclusters distributed in tissues.

File Name: Supplementary Data 4
Description: Major clusters and subclusters distributed in patients.

File Name: Supplementary Data 5
Description: Top50 markers of cell subclusters.

File Name: Supplementary Data 6
Description: The detailed gene signatures for cell function.

File Name: Supplementary Data 7
Description: Characteristics of patients and specimens included in experiments.

File Name: Supplementary Data 8
Description: The dynamic alterations in the top-scoring 50 genes of MP6 across different groups.

File Name: Supplementary Data 9
Description: HRR-associated genes identified in the whole-exome sequencing data.

File Name: Supplementary Data 10
Description: shRNA and sgRNA used in the study.
