# Supplementary material for: Hypoxia-driven remodeling of SELENOP+ macrophages shapes T cell dynamics and promotes ovarian cancer metastasis
Source: Nat Commun. 2026 Jan 12;17:1097. doi: 10.1038/s41467-025-67859-2 (PMC12852879; doi:10.1038/s41467-025-67859-2)
Supplement: Supplementary file 13 — Reporting Summary [file 41467_2025_67859_MOESM13_ESM.pdf]

nature portfolio

Corresponding author(s): Xin Zhou
Last updated by author(s): Nov 17, 2025

Reporting Summary

Nature Portfolio wishes to improve the reproducibility of the work that we publish. This form provides structure for consistency and transparency in reporting. For further information on Nature Portfolio policies, see our Editorial Policies and the Editorial Policy Checklist.

Statistics

For all statistical analyses, confirm that the following items are present in the figure legend, table legend, main text, or Methods section.

n/a	Confirmed	
☐	☒	The exact sample size (*n*) for each experimental group/condition, given as a discrete number and unit of measurement
☐	☒	A statement on whether measurements were taken from distinct samples or whether the same sample was measured repeatedly
☐	☒	The statistical test(s) used AND whether they are one- or two-sided<br>*Only common tests should be described solely by name; describe more complex techniques in the Methods section.*
☐	☒	A description of all covariates tested
☐	☒	A description of any assumptions or corrections, such as tests of normality and adjustment for multiple comparisons
☐	☒	A full description of the statistical parameters including central tendency (e.g. means) or other basic estimates (e.g. regression coefficient) AND variation (e.g. standard deviation) or associated estimates of uncertainty (e.g. confidence intervals)
☐	☒	For null hypothesis testing, the test statistic (e.g. *F*, *t*, *r*) with confidence intervals, effect sizes, degrees of freedom and *P* value noted<br>*Give P values as exact values whenever suitable.*
☐	☒	For Bayesian analysis, information on the choice of priors and Markov chain Monte Carlo settings
☒	☐	For hierarchical and complex designs, identification of the appropriate level for tests and full reporting of outcomes
☐	☒	Estimates of effect sizes (e.g. Cohen's *d*, Pearson's *r*), indicating how they were calculated

Our web collection on statistics for biologists contains articles on many of the points above.

Software and code

Policy information about availability of computer code

Data collection	Single-cell RNA sequencing libraries were prepared according to the 10x Genomics Chromium single cell 3' platform protocol, and the constructed libraries were sequenced on Illumina Novaseq 6000 sequencer. Cell Ranger software (v3.1.0) was used to collect raw scRNA-seq data. Stereo-seq raw data were automatically processed using the BGI Stereomics analytical pipeline. The libraries of WES were pooled and loaded on DNBseq-T7. FACS Diva Software (v8.0.2, BD) was conducted to collect FACS data.
Data analysis	Softwares were used as follows:<br>1. Cell Ranger (v3.1.0) was used to process single-cell sequencing data and align it to the GRCh38 human reference genome.<br>2. DoubletFinder (v2.0.1) was used to estimated and remove potential doublets.<br>3. Seurat (v4.3.0) was used to analyze single cell RNA-seq data.<br>4. Harmony (v1.0) was used to integrate multiple RNA-seq dataset and correct batch effect.<br>5. pcaExplorer (v2.14.2) was used for interactive visualization of RNA-seq data using a principal components approach.<br>6. UCell (2.5.0) was used for rank-based signature enrichment analysis for single-cell data.<br>7. Cell Ranger (v.7.1.0) was used for processing the raw data of TCR sequences.<br>8. Monocle2 (v2.30.0) was used to infer cell lineage developmental trajectory.<br>9. GSVA (v1.40.0) was used for signature enrichment analysis.<br>10. BayesPrism (v2.2.2) was used for Bayesian cell type and gene expression deconvolution.<br>11. cell2location (v0.1.0) was used to assess the spatial distribution of cell types.<br>12. mistyR (v1.8.1) was used to estimate the importance of the abundance of each cell type in explaining the abundance of the other major cell types.<br>13. pySCENIC (v0.12.1) was used to estimate transcription factor activity.<br>14. inferCNV (v1.10.1) was used to compute chromosomal copy number variations (CNV).

15. PROGENy (v1.20.0) was used to calculate 14 signaling pathways, whose scores were computed by a weighted sum of the product from expression and the weight of footprint genes.
16. CellPhoneDB (v3.0.0) was used to explore cell-cell interactions.
17. All analyses were performed on the following softwares: R (v4.2.2), Python (v3.9.0) and GraphPad Prism (v8.0.2).
18. BWA (version 0.7.17) was used to align the qualified reads from each sample to the assembled human reference genome (hg38).
19. FlowJo (v10.8.1) was used to analysed FACS data.
No novel algorithms were created for this study. All code used for analysis is available in GitHub (https://github.com/NCarticle2025/new).

For manuscripts utilizing custom algorithms or software that are central to the research but not yet described in published literature, software must be made available to editors and reviewers. We strongly encourage code deposition in a community repository (e.g. GitHub). See the Nature Portfolio guidelines for submitting code & software for further information.

Data

Policy information about availability of data

All manuscripts must include a data availability statement. This statement should provide the following information, where applicable:

- Accession codes, unique identifiers, or web links for publicly available datasets
- A description of any restrictions on data availability
- For clinical datasets or third party data, please ensure that the statement adheres to our policy

The sc-RNAseq, WES and ST data generated in this study have been deposited in the Genome Sequence Archive for Human (GSA-Human) database under accession code HRA006423 (https://ngdc.cncb.ac.cn/gsa-human/browse/HRA006423) and HRA003236 (https://ngdc.cncb.ac.cn/gsa-human/browse/HRA003236). The sc-RNAseq, WES and ST data are available under restricted access for reasons related to human genetic resources, access can be obtained by contacting the corresponding author (Xin Zhou, xzhou@cmu.edu.cn and Chaoyang Sun, suncydoctor@gmail.com). Raw data access requests will be processed within 3 months by following the guidelines for Genome Sequence Archive for noncommercial use. Data access will be granted for 1 year. The raw sc-RNAseq, WES and ST data are protected and are not available due to data privacy laws. The detailed data generated in this study are provided in the Supplementary Information/Source Data file. Source data are provided with this paper. The previously published data used in this study are available in the NCBI Gene Expression Omnibus (https://www.ncbi.nlm.nih.gov/gds/) under accession no. GSE184880, GSE203612, GSE266577; GSA-Human (https://ngdc.cncb.ac.cn/gsa-human/) under accession code HRA002184, HRA002767; Synapse (https://www.synapse.org/msk_spectrum) under accession number syn52458609; and https://doi.org/10.6084/m9.figshare.22147103.

Research involving human participants, their data, or biological material

Policy information about studies with human participants or human data. See also policy information about sex, gender (identity/presentation), and sexual orientation and race, ethnicity and racism.

Reporting on sex and gender	Sex was not considered a biological variable in the study, as ovarian cancer is a gender specific disease. Only female patients were included in our study. Patients' self-reported gender were used for the analysis.
Reporting on race, ethnicity, or other socially relevant groupings	The research was not involved in race, ethnicity, or other socially relevant groupings. The relevant information was not taken under consideration.
Population characteristics	The single-cell RNA sequencing cohort in our study consisted of seventeen female patients, and all patients were adults (age range: 40-78). Of these, nine donors were pathologically diagnosed benign uterine fibroids patients; two donors were pathologically diagnosed HGSOC patients at FIGO stage I-II ; six donors were pathologically diagnosed HGSOC patients at FIGO stage III-IV. Our scRNA-seq cohort included seven HGSOC patients with HRD-positive tumors, comprising four HGSOC patients with BRCA1/2-mutated tumors (three with germline mutations and one with somatic mutations) and three HGSOC patients with tumors harboring no alterations in BRCA1/2. The characteristics of the patients in the single-cell RNA sequencing cohort are detailed in Supplementary Data 1. The spatial transcriptomics cohort in our study consisted of additional six female patients pathologically diagnosed as metastatic HGSOC (age range: 41-56). The mIHC validation cohort consisted of twenty-four HGSOC patients (age range: 36-69). Additionally, one more HGSOC patients, aged 51, were enrolled for the generation of patient derived organoids for experimental validation.
Recruitment	Patients eligible for pathological diagnosis were recruited from ShengJing Hospital of China Medical University (Shenyang, China), the First Hospital of China Medical University (Shenyang, China), and Tongji Hospital (Wuhan, China), in accordance with ethical guidelines. A total of thirty-four samples were collected for the single-cell RNA sequencing cohort, including two adnexal tumors samples from two HGSOC patients at FIGO stage I-II (EAT Group); six adnexal tumors samples from six HGSOC patients at FIGO stage III-IV (LAT Group); nine matched metastatic sites samples, encompassing four omental metastases samples (Met.Ome Group) and five peritoneal metastases samples (Met.Per Group) from six HGSOC patients at FIGO stage III-IV; two matched peritoneal lavage fluid samples from two HGSOC patients at FIGO stage I-II (PLF.EOC Group); six matched ascites samples from six HGSOC patients at FIGO stage III-IV (Ascites Group); six peritoneal lavage fluid samples from six benign uterine fibroids patients (PLF.UF Group); three normal post-menopausal ovarian tissue samples from three benign uterine fibroids patients (Nor.Ovr Group). Twenty-four samples were collected from additional six HGSOC patients for spatial transcriptomics cohort, including eleven adnexal tumors, six omental metastases, and seven other metastatic samples. Thirty-four samples were collected from additional twenty-four HGSOC patients for mIHC validation cohort, including fourteen adnexal tumors, fifteen omental metastases, and five peritoneal metastases samples. There is no potential self-selection bias.
Ethics oversight	This study was performed following the ethical guidelines of the Declaration of Helsinki and was approved by the Research Ethics Committee of Shengjing Hospital of China Medical University, the Research Ethics Committee of Tongji Hospital, Tongji Medical College, Huazhong University of Science and Technology, and the Research Ethics Committee of the First Hospital of

China Medical University. Written informed consent was obtained from all patients involved in this study for the use of their tissue samples and clinical information and participant compensation was not implemented in this study.

Note that full information on the approval of the study protocol must also be provided in the manuscript.

Field-specific reporting

Please select the one below that is the best fit for your research. If you are not sure, read the appropriate sections before making your selection.

☒ Life sciences ☐ Behavioural & social sciences ☐ Ecological, evolutionary & environmental sciences

For a reference copy of the document with all sections, see nature.com/documents/nr-reporting-summary-flat.pdf

Life sciences study design

All studies must disclose on these points even when the disclosure is negative.

Sample size	Since samples used for scRNA-seq and ST analysis were human subjects, we collected as many samples as possible within our time frame. We used adequate numbers of samples and mice that would provide statistically significant results. The detailed information is: scRNA-seq transcriptomic profiles of total 34 samples were from 17 patients, including 2 adnexal tumors from two HGSOC patients at FIGO stage I-II (EAT Group), 6 adnexal tumors from six HGSOC patients at FIGO stage III-IV (LAT Group); 4 matched omental metastases (Met.Ome Group) and 5 matched peritoneal metastases (Met.Per Group) from six HGSOC patients at FIGO stage III-IV; 2 matched peritoneal lavage fluid from two HGSOC patients at FIGO stage I-II (PLF.EOC Group); 6 matched ascites from six HGSOC patients at FIGO stage III-IV (Ascites Group); 6 peritoneal lavage fluid from six benign uterine fibroids patients (PLF.UF Group); and 3 normal post-menopausal ovarian tissues from three benign uterine fibroids patients (Nor.Ovr Group). Additionally, the ST profiles were from 6 additional HGSOC patients, including 11 adnexal tumors, 6 omental metastases, and 7 other metastatic lesions. The mIHC validation cohort were from 24 additional HGSOC patients, including 14 adnexal tumors, 15 omental metastases, and 5 peritoneal metastases. Additional 1 HGSOC patients were recruited to culture patient derived organoids for experimental validations. No sample size calculation was performed.
Data exclusions	Low-quality cells and genes were removed based on several criteria: cells with fewer than 200 genes, fewer than 800 UMI counts, top 1% UMI count, over 20% mitochondrial gene content, and genes present in fewer than 3 cells. The "DoubletFinder" R package identified and removed potential doublets.
Replication	All replicates were successful, and the corresponding figure legends provide detailed information.
Randomization	The patients with HGSOC were recruited randomly in this study. This is an observational study, rather than a clinical trial, so no randomization was performed. In in vivo experiments, mice with comparable luminescence intensity were assigned to different groups.
Blinding	Exclusion of low-quality cells and genes were carried out based on predefined criteria before single-cell data analysis, irrespective of group assignment. Investigators involved in single-cell RNA library preparation and sequencing did not participate in data analysis. For the in vitro studies blinding was not relevant as all measures were quantified by standard cellular and biochemical assays. Key results were validated by 2 independent operators.

Reporting for specific materials, systems and methods

We require information from authors about some types of materials, experimental systems and methods used in many studies. Here, indicate whether each material, system or method listed is relevant to your study. If you are not sure if a list item applies to your research, read the appropriate section before selecting a response.

Materials & experimental systems

n/a	Involved in the study	
☐	☒	Antibodies
☐	☒	Eukaryotic cell lines
☒	☐	Palaeontology and archaeology
☐	☒	Animals and other organisms
☒	☐	Clinical data
☒	☐	Dual use research of concern
☒	☐	Plants

Methods

n/a	Involved in the study	
☒	☐	ChIP-seq
☐	☒	Flow cytometry
☒	☐	MRI-based neuroimaging

Antibodies

Antibodies used	Antibodies used for multiplex immunofluorescence:<br>Anti-CD8a, Abmart (Cat#PAQ6570), used at 1:300 dilution<br>Anti-PD1, Abmart (Cat#PH9964), used at 1:300 dilution<br>Anti-GZMH, Bioworld (Cat#BS2543), used at 1:300 dilution<br>Anti-SPP1, Abcam (Cat#ab63856), used at 1:200 dilution<br>Anti-SELENOP, Invitrogen (Cat#PA5-112707), used at 1:200 dilution

Anti-HIF-1α, Abmart (Cat#PU774605), used at 1:200 dilution
Anti-CD68, Abcam (Cat#ab283654), used at 1:500 dilution
Anti-Panck, MXB Biotechnoiogies (Cat#RAB-0050), used at 1:10 dilution

Antibodies used for flow cytometry:
Anti-CD45.1-PE-Cy7 monoclonal antibody, Thermo (Cat#25-0453-81), used at 0.5 μg/test
Anti-CD45.2-AF700 monoclonal antibody, BioLegend (Cat#147716), used at 0.25 μg/test
Anti-CD3e-BV421 monoclonal antibody, Invitrogen (Cat#48-0031-82), used at 0.5 μg/test
Anti-CD8a-BV480 monoclonal antibody, Invitrogen (Cat#69-0081-82), used at 0.5 μg/test
Anti-F4/80-BV650 monoclonal antibody, Thermo (Cat#416-4801-82), used at 0.5 μg/test
Anti-CD11b-PerCP-Cy5.5 monoclonal antibody, BD Pharmingen (Cat#550993), used at 0.5 μg/test
Anti-GZMB-PE-Cy7 monoclonal antibody, Thermo (Cat#25-8898-82), used at 1 μg/test
Anti-GZMB-PE monoclonal antibody, BioLegend (Cat#372208), used at 1 μg/test
anti-PRF1 monoclonal antibody, BioLegend (Cat#154304), used at 1 μg/test
anti-PRF1 monoclonal antibody, Invitrogen (Cat#17-9392-80), used at 1 μg/test
anti-PD-1 monoclonal antibody, Biolegend (Cat#135225), used at 1 μg/test
anti-SPP1 monoclonal antibody, SantaCruz (Cat#sc-73631PE), used at 1 μg/test
anti-SELENOP antibody, SantaCruz (Cat#sc-376858FITC), used at 1 μg/test
anti-CD86 monoclonal antibody, Invitrogen (Cat#63-0862-80), used at 0.5 μg/test
anti-CD206 monoclonal antibody, Invitrogen (Cat#17-2061-80), used at 0.5 μg/test

Antibodies used for Western Blot:
Anti-GPX1 antibody, Abmart (Cat#T56586S), used at 1:1000 dilution
Anti-SELENOP, Invitrogen (Cat#PA5-112707), used at 1:900 dilution
Anti-SELENOP, SantaCruz (Cat#sc-376858), used at 1:1000 dilution
Anti-SELENOP, Abcam (Cat#ab277526), used at 1:1000 dilution
Anti-EPHB2, Proteintech (Cat#83277-1-RR), used at 1:1000 dilution
Anti-VEGFA, BioLegend, (Cat#512810), used at 1:1000 dilution
Anti-GAPDH, Abcam, (Cat#ab8245), used at 1:5000 dilution
Anti-β-actin antibody, Proteintech (Cat#66009-1-Ig), used at 1:5000 dilution
Goat anti-rabbit IgG-HRP antibody, Absin (Cat# abs20040), used at 1:5000 dilution
Goat anti-mouse IgG-HRP antibody, Absin (Cat#abs20039), used at 1:5000 dilution

Cancer cells and macrophages coculture system
anti-human/mouse VEGFA neutralizing antibody (Cat#HY-P9906, MedChemExpress; Cat# 512810, BioLegend), used at 50 ug/mL

Validation

All of the antibodies used in this study were validated for mIHC, FACS and Western Blot use in human specimens by both the manufacturers and our pre-experiment. The detailed antibody information and antibody validation procedures were described on the following respective manufacturers' websites: https://www.ab-mart.com.cn, https://bioworlde.com, https://www.abcam.cn, https://www.thermofisher.cn, https://www.crunchbase.com, https://www.biolegend.com, https://www.bdbiosciences.com/zh-cn, and https://www.scbt.com, https://www.ptgcn.com.

Eukaryotic cell lines

Policy information about cell lines and Sex and Gender in Research

Cell line source(s)

The human ovarian cancer cell line OVCAR3 and CAOV3 was bought from Wuhan Pricella Biotechnology Co., Ltd (CL-0178). The human ovarian cancer cell line COV362 were purchased from GuangZhou Jennio Biotech Co., Ltd. The mouse ovarian cancer cell line ID8 was provided by X.C. (China Medical University). THP-1 cells were provided by Y.S. (China Medical University).

Authentication

The cell lines used in this study were authenticated with STR profiling.

Mycoplasma contamination

The cell lines used in this study were confirmed to be mycoplasma negative.

Commonly misidentified lines
(See ICLAC register)

No any commonly misidentified cell lines were used in this study.

Animals and other research organisms

Policy information about studies involving animals; ARRIVE guidelines recommended for reporting animal research, and Sex and Gender in Research

Laboratory animals

OT-1 (C57BL/6-Tg (TcraTcrb)1100Mjb/J) and CD45.1 (B6.SJL-PtprcaPepcb/BoyJ) were generously provided by the First Hospital of China Medical University. C57BL/6J (B6) mice were obtained from GemPharmatech Co., Ltd. Age-matched (6-10 weeks) female mice were used in all mouse experiments. All the mice were raised in an SPF environment with a temperature of 24℃, a relative humidity of 50% to 60%, and a 12-hour light/12-hour dark cycle.

Wild animals

Wild animal were not involved in the study.

Reporting on sex

Age-matched (6-10 weeks) female mice were used in all mouse experiments. Female mice were selected because ovarian cancer is a gender specific disease.

Field-collected samples | No field-collected samples are involved in the study.

Ethics oversight | All animal studies were reviewed and approved by the Ethics Committee of the Laboratory Animal Department of China Medical University. Measures were taken to minimize animal distress. According to institutional policies on tumor production, the maximum allowable tumor diameter in any direction is 2.0 cm, and this limit was not exceeded in any of our experiments.

Note that full information on the approval of the study protocol must also be provided in the manuscript.

Plants

Seed stocks | Not applicable.

Novel plant genotypes | Not applicable.

Authentication | Not applicable.

Flow Cytometry

Plots

Confirm that:

☒ The axis labels state the marker and fluorochrome used (e.g. CD4-FITC).

☒ The axis scales are clearly visible. Include numbers along axes only for bottom left plot of group (a 'group' is an analysis of identical markers).

☒ All plots are contour plots with outliers or pseudocolor plots.

☒ A numerical value for number of cells or percentage (with statistics) is provided.

Methodology

Sample preparation | For cells grown in vitro: Cells grown in vitro from each group were first stimulated with the cell stimulation cocktail (Cat#00-4975-93, Invitrogen) for 5 h. Next, cells were stained with eBioscience™ Fixable Viability Dye eFluor™ 780 (Cat#65-0865-14, Invitrogen) or Zombie Red™ Fixable Viability Kit (Cat#423109, Biolegend), blocking with anti-mouse CD16/32 (Cat#101320, BioLegend), or Human TruStain FcX™ (Cat#422302, Biolegend), and staining for 30 min at 4℃. Cells were stained with the anti-mouse/human antibody and fixed/permeabilized using the Cytofix/Cytoperm™ Fixation/ Permeabilization Kit (Cat#554714, BD Biosciences). Cells were resuspended in PBS with 1% FBS for flow cytometry analysis.
For cells from tumor tissue: Tumor tissue was minced then digested in a digestion solution (1 M HEPES, 1 mg/ml collagenase I, 1 mg/ml collagenase IV, 0.2 mg/ml DNase I, 0.2 mg/ml Hyaluronidase, 1% Penicillin-streptomycin complex and 10% FBS). The dissociated tissues were filtered through 70 μm strainers to obtain single cells. Cells from each group were stimulated with the cell stimulation cocktail (Cat#00-4975-93, Invitrogen) for 5 h. Next, cells were stained with eBioscience™ Fixable Viability Dye eFluor™ 780 (Cat#65-0865-14, Invitrogen) or Zombie Red™ Fixable Viability Kit (Cat#423109, BioLegend), blocking with anti-mouse CD16/32 (Cat#101320, BioLegend) and staining for 30 min at 4℃. Cells were stained with the anti-mouse antibody and fixed/permeabilized using the Cytofix/Cytoperm™ Fixation/Permeabilization Kit (Cat#554714, BD Biosciences). Cells were resuspended in PBS with 1% FBS for flow cytometry analysis.

Instrument | BD FACSymphony A1

Software | BD FACS Diva Software (version 8.0.2, BD)

Cell population abundance | In T Cell Isolation and SELENOP protein-supply assay: live cell was ~51.8% of single cell population; CD3e+ CD8a+ cell (OT-I T cell) was ~87.3% of the live cell population.
In T cell Killing Assay: live cell was ~74.4% of single cell population; CD3e+ CD8a+ cell (CTL) was ~92.5% of the live cell population.
In Animal studies: live cell was ~69.2% of single cell population; CD3e+ CD8a+ cell (CD8+ T cell) was ~7.0% of the live cell population. F4/80+ CD11b+ cell (macrophage) was ~4.1% of the live cell population.

Gating strategy | SSC-A and FSC-A were used to gate single cells; eBioscience™ Fixable Viability Dye eFluor™ 780 or Zombie Red™ Fixable Viability were used to gate APC-Cy7- or PI- alive cells; CD45 was used to gate immune cells; CD3e and CD8a were used to gate CD8+ T cells; F4/80 and CD11b were used to gate macrophages.

☒ Tick this box to confirm that a figure exemplifying the gating strategy is provided in the Supplementary Information.
